# Supplementary material for: Dose–Response Associations Between Diet and Risk of Rheumatoid Arthritis: A Meta-Analysis of Prospective Cohort Studies
Source: Nutrients. 2024 Nov 26;16(23):4050. doi: 10.3390/nu16234050 (PMC11644016; doi:10.3390/nu16234050)
Supplement: Supplementary file 1 [file nutrients-16-04050-s001.zip › nutrients-3306803-supplementary.pdf]

Dose-response associations between diet and risk of Rheumatoid Arthritis: a meta-analysis of prospective cohort studies

Additional file 1

<u>Supplemental Tables:</u>

Table S1 Search strategy
Table S2 PICOS criteria for inclusion and exclusion of studies
Table S3 Standard conversions for food groups and nutrients
Table S4 Studies reviewed in full text for eligibility (excluded reasons for meta-analysis)
Table S5 Characteristics of the included studies on the consumption of food, and nutrient and risk of rheumatoid arthritis
Table S6 Characteristics of 14 included cohort studies
Table S7 (a). Relationship between food groups and beverages consumption and risk of rheumatoid arthritis: a pooled analysis comparing the highest with the lowest categories and dose-response analyses
Table S7 (b). Relationship between nutrient consumption and risk of RA: a pooled analysis comparing the highest with the lowest categories and dose-response analyses
Table S8. Scoring for the food groups, beverages and food components of NutriGrade for the risk of rheumatoid arthritis
Table S9. Quality assessment of cohort studies included in meta-analysis (Newcastle-Ottawa Quality Assessment Scale)
Table S10 (a). Subgroup analyses for total alcohol consumption and risk of rheumatoid arthritis
Table S10 (b). Subgroup analyses for total alcohol consumption and risk of seropositive rheumatoid arthritis
Table S10 (c). Subgroup analyses for total coffee consumption and risk of rheumatoid arthritis
Table S10 (d). Subgroup analyses for decaffeinated coffee consumption and risk of rheumatoid arthritis
Table S10 (e). Subgroup analyses for tea consumption and risk of rheumatoid arthritis
Table S10 (f). Subgroup analyses for fruit consumption and risk of rheumatoid arthritis
Table S10 (g). Subgroup analyses for vegetable consumption and risk of rheumatoid arthritis
Table S11 (a). Sensitivity analyses for food group consumption and risk of rheumatoid arthritis: excluding one study at a time
Table S11 (b). Sensitivity analyses for nutrient intake and risk of rheumatoid arthritis: excluding one study at a time
Table S12 (a). Trim and fill tests for food group consumption and risk of rheumatoid arthritis
Table S12 (b). Trim and fill tests for nutrients consumption and risk of rheumatoid arthritis

Supplemental Figures:

Figure S1. Non-linear dose-response meta-analyses for food group, beverages and risk of rheumatoid arthritis using restricted cubic splines
Figure S2. Non-linear dose-response meta-analyses for nutrient intake and risk of rheumatoid arthritis using restricted cubic splines
Figure S3. Linear dose-response analyses for alcohol consumption and risk of rheumatoid arthritis
Figure S4. Meta-analysis of total alcohol consumption and risk of seropositive rheumatoid arthritis comparing highest with the lowest categories
Figure S5. Linear dose-response analyses for total alcohol consumption and risk of seropositive rheumatoid arthritis
Figure S6. Meta-analysis of non-alcoholic beverages (tea and coffee, soda) consumption and risk of rheumatoid arthritis comparing highest with the lowest categories
Figure S7. Linear dose-response analyses for non-alcoholic beverages (tea and coffee, soda) consumption and risk of rheumatoid arthritis
Figure S8. Meta-analysis of tea, and decaffeinated coffee consumption and risk of RF-positive rheumatoid arthritis comparing highest with the lowest categories
Figure S9. Linear dose-response analyses for decaffeinated coffee, and tea consumption and risk of RF-positive rheumatoid arthritis
Figure S10. Meta-analysis of fruit, and vegetable consumption and risk of rheumatoid arthritis comparing highest with the lowest categories
Figure S11. Linear dose-response analyses for fruit, and vegetable consumption and risk of rheumatoid arthritis
Figure S12. Meta-analysis of meat, and fish consumption and risk of rheumatoid arthritis comparing highest with the lowest categories
Figure S13. Linear dose-response analyses for meat, and fish consumption and risk of rheumatoid arthritis
Figure S14. Meta-analysis of other food component consumption and risk of rheumatoid arthritis comparing highest with the lowest categories
Figure S15. Linear dose-response analyses for other food group consumption and risk of rheumatoid arthritis
Figure S16. Meta-analysis of dietary n-3 PUFA and risk of rheumatoid arthritis comparing highest with the lowest categories
Figure S17. Linear dose-response analyses for dietary n-3 PUFA and risk of rheumatoid arthritis
Figure S18. Meta-analysis of vitamins intake and risk of rheumatoid arthritis comparing highest with the lowest categories
Figure S19. Linear dose-response analyses for vitamins intake and risk of rheumatoid arthritis
Figure S20. Meta-analysis of dietary carotenoids consumption and risk of rheumatoid arthritis comparing highest with the lowest categories
Figure S21. Linear dose-response analyses for carotenoids consumption and risk of rheumatoid arthritis
Figure S22. Funnel plots for food group, and beverages and risk of rheumatoid arthritis comparing extreme the highest with the lowest groups
Figure S23. Funnel plots for nutrient intake and risk of rheumatoid arthritis comparing extreme the highest with the lowest groups

Table S1. Search strategy

Ovid Medline strategy:

1	(food* or nutrition or nutrient* or diet* or meat or red meat or processed meat or meat consumption* or meat product* or fish consumption* or dietary fish or fish oil or fruit* or milk or dairy product* or soda consumption* or dietary sucrose or energy intake* or protein consumption* or dietary protein or calcium or coffee consumption* or Caffeine or Olive oil or food component* or Zinc or dietary pattern* or Mediterranean diet* or dietary approaches to stop hypertension or DASH diet* or Inflammatory dietary pattern* or eating pattern* or western diet* or long-chain n-3 polyunsaturated fatty acid* or PUFA* or omega-3 fatty acid* or fatty acid* or low-carbohydrate diet* or micronutrient*).tw.
2	(meat or "dairy product" or "dietary carbohydrates OR dietary proteins OR dietary supplements" or "fast food" or honey or nutrients or eggs or "diet, food ,and nutrition" or "food and beverages" or "DASH diet OR dietary approaches to stop hypertension" or "functional food" or micronutrients or "Mediterranean diet" or "energy intake" or "high-fat diet" or "western diet" or "Diabetic Diet" or "High-Protein Diet").tw.
3	(vegetable* or vegan or vegan diet* or vegetarian or vegetarian diet*).tw.
4	(vegetables or vegan or vegetarian or "vegan diet" or "vegetarian diet").tw.
5	(alcohol* or alcohol use* or alcohol intake* or alcohol exposure or alcohol consumption* or alcohol drinking).tw.
6	(alcohols or "alcoholic beverages" or "alcohol drinking").tw.
7	(antioxidant* or antioxidant intake*).tw.
8	antioxidants.tw.
9	(dietary fiber or dietary fibre or high-fiber dietary or fiber intake* or fibre intake* or probiotics).tw.
10	("dietary fiber" or probiotics).tw.
11	("vitamin K" or "vitamin E" or "vitamin D" or "vitamin B" or "vitamin B complex" or "vitamin A").tw.
12	(vitamin or vitamin intake* or vitamin B group or vitamin D or vitamin B6 or vitamin B12 or vitamin K or vitamin D deficiency or vitamin K2 or vitamin E).tw.
13	or/1-12
14	(("Rheumatoid arthritis" or "inflammatory arthritis").tw. or (Rheumatoid Arthritis.tw. or Arthritis, Rheumatoid/)) not treatment.tw.
15	randomized controlled trial.pt.
16	(random$ or placebo$ or single blind$ or double blind$ or triple blind$).ti,ab.
17	(retraction of publication or retracted publication).pt.
18	or/15-17
19	(animals not humans).sh.
20	((comment or editorial or meta-analysis or practice-guideline or review or letter) not "randomized controlled trial").pt.
21	(random sampl$ or random digit$ or random effect$ or random survey or random regression).ti,ab. not "randomized controlled trial".pt.
22	18 not (19 or 20 or 21)
23	exp cohort studies/
24	cohort$.tw.
25	controlled clinical trial.pt.
26	epidemiologic methods/
27	limit 26 to yr=1971-1988
28	or/23-25,27
29	22 or 28

30	13 and 14 and 29
31	limit 30 to (english language and humans)

Ovid Embase strategy:

1	(food* or nutrition or nutrient* or diet* or meat or red meat or processed meat or meat consumption* or meat product* or fish consumption* or dietary fish or fish oil or fruit* or milk or dairy product* or soda consumption* or dietary sucrose or energy intake* or protein consumption* or dietary protein or calcium or coffee consumption* or Caffeine or Olive oil or food component* or Zinc or dietary pattern* or Mediterranean diet* or dietary approaches to stop hypertension or DASH diet* or Inflammatory dietary pattern* or eating pattern* or western diet* or long-chain n-3 polyunsaturated fatty acid* or PUFA* or omega-3 fatty acid* or fatty acid* or low-carbohydrate diet* or micronutrient*).tw.
2	(meat or "dairy product" or "dietary carbohudratesOR dietary proteinsOR dietary supplements" or "fast food" or honey or nutrients or eggs or "diet,food,and nutrition" or "food and beverages" or "DASH dietOR dietary approaches to stop hypertension" or "functional food" or micronutrients or "Mediterranean diet" or "energy intake" or "high-fat diet" or "western diet" or "Diabetic Diet" or "High-Protein Diet").tw.
3	(vegetable* or vegan or vegan diet* or vegetarian or vegetarian diet*).tw.
4	(vegetables or vegan or vegetarian or "vegan diet" or "vegetarian diet").tw.
5	(alcohol* or alcohol use* or alcohol intake* or alcohol exposure or alcohol consumption* or alcohol drinking).tw.
6	(alcohols or "alcoholic beverages" or "alcohol drinking").tw.
7	(antioxidant* or antioxidant intake*).tw.
8	antioxidants.tw.
9	(dietary fiber or dietary fibre or high-fiber dietary or fiber intake* or fibre intake* or probiotics).tw.
10	("dietary fiber" or probiotics).tw.
11	("vitamin K" or "vitamin E" or "vitamin D" or "vitamin B" or "vitamin B complex" or "vitamin A").tw.
12	(vitamin or vitamin intake* or vitamin B group or vitamin D or vitamin B6 or vitamin B12 or vitamin K or vitamin D deficiency or vitamin K2 or vitamin E).tw.
13	or/1-12
14	(("Rheumatoid arthritis" or "inflammatory arthritis").tw. or (Rheumatoid Arthritis.tw. or Arthritis, Rheumatoid/)) not treatment.tw.
15	(random$ or placebo$ or single blind$ or double blind$ or triple blind$).ti,ab.
16	RETRACTED ARTICLE/
17	or/15-16
18	(animal$ not human$).sh,hw.
19	(book or conference paper or editorial or letter or review).pt. not exp randomized controlled trial/
20	(random sampl$ or random digit$ or random effect$ or random survey or random regression).ti,ab. not exp randomized controlled trial/
21	17 not (18 or 19 or 20)
22	exp cohort analysis/
23	exp longitudinal study/
24	exp prospective study/
25	exp follow up/
26	cohort$.tw.
27	or/22-26
28	21 or 27
29	13 and 14 and 28

30	limit 29 to (human and english language and yr="2000 -Current")

Web of Science strategy:

1	TS = (food* or nutrition or nutrient* or diet* or meat or red meat or processed meat or meat consumption* or meat product* or fish consumption* or dietary fish or fish oil or fruit* or milk or dairy product* or soda consumption* or dietary sucrose or energy intake* or protein consumption* or dietary protein or calcium or coffee consumption* or Caffeine or Olive oil or food component* or Zinc or dietary pattern* or Mediterranean diet* or dietary approaches to stop hypertension or DASH diet* or Inflammatory dietary pattern* or eating pattern* or western diet* or long-chain n-3 polyunsaturated fatty acid* or PUFA* or omega-3 fatty acid* or fatty acid* or low-carbohydrate diet* or micronutrient*)
2	TS= (meat or "dairy product" or "dietary carbohudratesOR dietary proteinsOR dietary supplements" or "fast food" or honey or nutrients or eggs or "diet,food,and nutrition" or "food and beverages" or "DASH dietOR dietary approaches to stop hypertension" or "functional food" or micronutrients or "Mediterranean diet" or "energy intake" or "high-fat diet" or "western diet" or "Diabetic Diet" or "High-Protein Diet")
3	TS= (Vegetable* or vegan or vegan diet* or vegetarian or vegetarian diet*)
4	TS= (vegetables or vegan or vegetarian or "vegan diet" or "vegetarian diet")
5	TS= (alcohol* or alcohol use* or alcohol intake* or alcohol exposure or alcohol consumption* or alcohol drinking)
6	TS= (alcohols or "alcoholic beverages" or "alcohol drinking")
7	TS= (antioxidant* or antioxidant intake*)
8	TS= antioxidants
9	TS= (dietary fiber or dietary fibre or high-fiber dietary or fiber intake* or fibre intake* or probiotics)
10	TS= ("dietary fiber" or probiotics)
11	TS= ("vitamin K" or "vitamin E" or "vitamin D" or "vitamin B" or "vitamin B complex" or "vitamin A")
12	TS= (vitamin or vitamin intake* or vitamin B group or vitamin D or vitamin B6 or vitamin B12 or vitamin K or vitamin D deficiency or vitamin K2 or vitamin E)
13	#12 OR #11 OR #10 OR #9 OR #8 OR #7 OR #6 OR #5 OR #4 OR #3 OR #2 OR #1
14	TS=((("Rheumatoid arthritis" or "inflammatory arthritis") or (Rheumatoid Arthritis or Arthritis, Rheumatoid/)) not treatment)
15	TS=(randomized controlled trial)
16	TS=(random$ or placebo$ or single blind$ or double blind$ or triple blind$)
17	TS=(retraction of publication or retracted publication)
18	#17 OR #16 OR #15
19	TS=(animals not humans)
20	TS=((comment or editorial or meta-analysis or practice-guideline or review or letter) not "randomized controlled trial")
21	TS=((random sampl$ or random digit$ or random effect$ or random survey or random regression) not "randomized controlled trial")
22	#18 NOT (#19 OR #20 OR #21)
23	TS=(cohort analysis OR longitudinal study OR prospective study OR follow up OR cohort$)
24	#23 OR #22
25	#13 AND #14 AND #24
26	((#25) AND PY=(2000-2024)) AND LA=(English)

Table S2. PICOS criteria for inclusion and exclusion of studies

	Inclusion criteria	Exclusion criteria
**Population**	General population of adults	Aged <18 years
**Intervention / Exposure**	Dietary intakes of Food component or Nutrient	No dietary exposure
**Comparison**	Relative Risk (RRs), or Hazard Ratios (HRs), or Incidence Risk Ratios (IRRs)	Other risk estimates
**Outcomes**	Risk of rheumatoid arthritis	Risk of other arthritis, other autoimmune disease
**Study type**	Prospective cohort studies	Cross-sectional, case-control, ecological, retrospective observational studies, clinical trials, and non-human studies

Table S3 Standard conversions for food groups and nutrients

Food components	Standard amount
Total alcohol/beer/wine/liquor	1 unit = 8 grams
	1 glass = 1 serving = 15 grams
Total coffee/caffeinated coffee/decaffeinated coffee/tea	1 cup = 1 serving
Sugar-sweetened soda	1 drink = 1 serving
Fruits/vegetables	1 portion = 1 serving = 80 grams
Total meat/red meat/processed meat/poultry	1 serving = 90 grams
Total fish/oily fish	1 serving = 100 grams
Total dairy/milk/cheese	1 serving = 1 cup
Legumes	1 serving = 80 grams
Cereal products	1 serving = 30 grams
Vitamin D	400 IU
Vitamin C	40 mg
Vitamin A	600 IU
Vitamin E	3 mg
N-3 PUFA	100 mg
α -carotenoid/ β -carotenoid/ β -cryptoxanthin/lycopene/Lutein/zeaxanthin	200 mg

Table S4. Studies reviewed in full text for eligibility (excluded reasons for meta-analysis)

No.	Results from full text review	Reasons for excluded
1	Samira Alizadeh, Amir Ghorbanihaghjo, Sousan Kolahi, et al.<br>Effect of omega-3 fatty acids supplementation on circulating osteoprotegerin ligand to osteoprotegerin ratio in female patients with rheumatoid arthritis,<br>Clinical Biochemistry, 2011; 44(13):S142.	conference abstract.
2	Sofia Charneca, Margarida Ferro, João Vasques, et al. Adherence to the mediterranean diet is associated with patient-reported functional status in rheumatoid arthritis. Clinical Nutrition, 2021; 9:356.	conference abstract.
3	Sigrun Chrubasik. Vitamin E for rheumatoid arthritis or osteoarthritis: low evidence of effectiveness. Zeitschrift für Rheumatologie.2003;62(5):491	review.
4	Cooles FAH, Pratt AG, Ng WF, et al. The effect of vitamin D on early rheumatoid arthritis: A retrospective cohort analysis. Arthritis & Rheumatism, 2013;131:578-579.	conference abstract.
5	Hahn, J., Malspeis, S., Choi, M.Y., et al. Association of Healthy Lifestyle Behaviors and the Risk of Developing Rheumatoid Arthritis Among Women. Arthritis Care Res, 2023;75: 272-276.	exposure is not dietary consumption.
6	Schernhammer E S, Hu F B, Giovannucci E, et al. Sugar-sweetened soft drink consumption and risk of pancreatic cancer in two prospective cohorts. Cancer Epidemiology Biomarkers & Prevention, 2005, 14(9): 2098-2105.	conference abstract.
7	Lahiri M, Luben RN, Morgan C, et al. Using lifestyle factors to identify individuals at higher risk of inflammatory polyarthritis (results from the European Prospective Investigation of Cancer-Norfolk and the Norfolk Arthritis Register--the EPIC-2-NOAR Study). Ann Rheum Dis. 2014;73(1):219-26.	conference abstract.
8	Liu C, Meng X, Zhang H, et al. Early-life famine exposure and rheumatoid arthritis in Chinese adult populations: a retrospective cohort study. BMJ Open. 2021;11(7):e043416.	exposure is not dietary consumption.

9	Maria López Lasanta, et al. Assessing dietary habits in a large cohort of rheumatoid arthritis and psoriatic arthritis patients: Results of the Spanish imid consortium. American college of rheumatology, 2014.	conference abstract.
10	Sparks JA, Barbhaiya M, Tedeschi SK,et al. Inflammatory dietary pattern and risk of developing rheumatoid arthritis in women. Clin Rheumatol. 2019;38(1):243-250.	exposure is not dietary consumption.
11	Rosell M, Wesley AM, Rydin K, et al. Dietary fish and fish oil and the risk of rheumatoid arthritis. Epidemiology. 2009;20(6):896-901.	case-control study.
12	Sparks JA, Chen CY, Hiraki LT,et al. Contributions of familial rheumatoid arthritis or lupus and environmental factors to risk of rheumatoid arthritis in women: a prospective cohort study. Arthritis Care Res (Hoboken). 2014;66(10):1438-46.	exposure is not dietary consumption.
13	Yang WM, Lv JF, Wang YY, et al. The Daily Intake Levels of Copper, Selenium, and Zinc Are Associated with Osteoarthritis but Not with Rheumatoid Arthritis in a Cross-sectional Study. Biol Trace Elem Res. 2023.	cross-sectional study.
14	Cai B, Zhou M, Xiao Q, et al. L-shaped association between serum 25-hydroxyvitamin D and all-cause mortality of individuals with rheumatoid arthritis. Rheumatology (Oxford). 2023 Feb 1;62(2):575-582.	exposure is not dietary consumption.
15	De Vito R, Fiori F, Ferraroni M, et al. Olive Oil and Nuts in Rheumatoid Arthritis Disease Activity. Nutrients. 2023;15(4):963.	cross-sectional study.
16	Pengpid S, Peltzer K. Prevalence and associated factors of cross-sectional and incident self-reported arthritis or rheumatism among a national community sample of middle-aged and older adults in Thailand. Front Public Health. 2023;11:1064751.	cross-sectional study.
17	Carubbi F, Alunno A, Mai F, et al. Adherence to the Mediterranean diet and the impact on clinical features in primary Sjögren's syndrome. Clin Exp Rheumatol. 2021;39 Suppl 133(6):190-196.	outcome is not RA.
18	VanEvery H, Yang W, Su J, et al. Low-Density Lipoprotein Cholesterol and the Risk of Rheumatoid Arthritis: A Prospective Study in a Chinese Cohort. Nutrients. 2022;14(6):1240.	exposure is not dietary consumption.

19	Liang X, Chou OHI, Cheung CL,et al. Is hypertension associated with arthritis? The United States national health and nutrition examination survey 1999-2018. Ann Med. 2022;54(1):1767-1775.	exposure is not dietary consumption.
20	Crowson CS, Gunderson TM, Dykhoff HJ, et al. Comprehensive assessment of multimorbidity burden in a population-based cohort of patients with rheumatoid arthritis. RMD Open. 2022;8(1):e002022.	exposure is not dietary consumption.
21	Guan T, Wu Z, Xu C, et al. The association of trace elements with arthritis in US adults: NHANES 2013-2016. J Trace Elem Med Biol. 2023;76:127122.	exposure is not dietary consumption.
22	Moradi A, Nezamoleslami S, Nezamoleslami S, et al. The association between dietary total antioxidant capacity with risk of rheumatoid arthritis in adults: A case-control study. Clin Nutr ESPEN. 2022;51:391-396.	case-control study.
23	Liu L, Xie S. Dietary fiber intake associated with risk of rheumatoid arthritis among U.S. adults: NHANES 2010-2020. Medicine (Baltimore). 2023;102(12):e33357.	cross-sectional study.
24	Kronzer VL, Lennon RJ, Sparks JA, Myasoedova E, Davis JM, Crowson CS. Association between work physical activity, dietary factors, and risk of rheumatoid arthritis. Semin Arthritis Rheum. 2022;57:152100.	case-control study.
25	Hatami E, Aghajani M, Pourmasoumi M, Haeri F, Boozari B, Nezamoleslami S, Clark CCT, Nezamoleslami S, Ghiasvand R. The relationship between animal flesh foods consumption and rheumatoid arthritis: a case-control study. Nutr J. 2022;30;21(1):51.	case-control study.

Table S5. Characteristics of the included studies on the consumption of food, and nutrient and risk of rheumatoid arthritis

Author, year (cohort study)	Country, recruitment,sex , baseline age(yrs), follow-up(yrs)	Case ascertainment	Cohort size(No of cases), Dietary assessment(No of FFQ item)	Dietary exposure	Adjustment for confounding variables
Cerhan 2003(IWHS)[1]	US, female,55-69,11	Diagnosis by one rheumatologist using ACR criteria	n=29 368(152), validated semi-quantitative FFQ	vitamin C, vitamin E, carotenoids, α -carotenoid, β -carotenoid, β -cryptoxanthin, lycopene, lutein + zeaxanthin, zinc,copper, selenium supplements, manganese, all fruits,citrus fruits, oranges, orange juice,grapefruit,grapefruit juice, all vegetables, green leafy vegetables, yellow vegetables, vegetables, cabbages, cauliflower,legumes, broccoli	1, 5, 9, 15, 16, 21, 36, 38
Costenbader 2008(NHS & NHSII)[2]	NHS:US,female, 30-55,22 NHSII:US,female,25-42,12	Diagnosis by two rheumatologists using ACR criteria	NHS: n=91 739(559), NHSII: n=94 650(163), validated semi-quantitative FFQ	vitamin D	3, 4, 5, 8, 12, 15, 16, 17, 34, 43
Costenbader 2010(NHS & NHSII)[3]	NHS:US,female, 30-55,24 NHSII:US,female,25-42,12	Diagnosis by two rheumatologists using ACR criteria	NHS: n=90 721(619), NHSII: n=93 922(168), validated semi-quantitative FFQ	vitamin A, vitamin C,vitamin E, α -carotenoid, β -carotenoid, β -cryptoxanthin, lycopene, lutein/zeaxanthin	3, 4, 5, 12, 15, 16
Di Giuseppe 2012(SMC)[4]	Sweden,female,54-89,6	Outpatient Register and the Swedish Rheumatology Register	n=34 141(197), validated FFQ	alcohol,beer,wine,liquor	1, 4, 5, 6, 15,17, 23, 47

Sundström 2019(SMC)[5]	Sweden,female,54-8,12	National Patient Register and the Swedish Rheumatology Register	n=35 600(368), validated FFQ	Meat(overall), red meat, processed meat, poultry, total dairy, milk,cheese	1, 5, 14, 21, 23, 24
Di Giuseppe 2014(SMC)[6]	Sweden,female,54-8,7.5	National Patient Register and the Swedish Rheumatology Register	n=32 232(205), validated FFQ	long-chain n-3 PUFAs, fish	1, 5, 14, 21, 22
Hedenstierna 2021(SNMC)[7]	Sweden,both sexes,51.5±15.9, 18	Swedish National Patient Registers	n=41 068(577), questionnaire for epidemiological research	alcohol	2, 4, 5, 6, 12, 27
Hiraki 2012(NHS & NHSII)[8]	NHS:US,female, 30-55,30 NHSII:US,female,25-42,18	Diagnosis by two rheumatologists using ACR criteria	NHS:n=73 629(652), NHSII:n=45 544(148), validated FFQ	vitamin D	1, 3, 4, 5, 12, 14, 15, 21, 31, 32, 33, 35
Merlino 2004(IWHS)[9]	US,female,55-69,11	Diagnosis by two rheumatologists using ACR criteria	n=29 368(152), validated semi-quantitative FFQ	total vitamin D, dietary vitamin D, supplemental vitamin D, milk products, butter, margarine, skim milk, whole milk	1, 5, 21, 30, 44
Lahiri 2012(EPIC-Norfolk Study)[10]	UK, both sexes, 40-79, 4	1987 ACR criteria	n=25 271(138), validated semi-quantitative FFQ	alcohol	4, 5, 6, 17, 27, 31, 43
Nguyen 2020(E3N-EPIC Cohort Study)[11]	France, female, 52.5±6.5, 10	1987 ACR criteria	n=62 629(480), validated FFQ	raw vegetables, legumes,fruits, cereal products, fish, meat, dairy products, unsaturated fat, alcohol, MD	1, 4, 5, 6, 11, 12, 21
Hu 2017(NHS & NHSII)[12]	NHS:US,female, 30-55,26 NHSII:US,female,25-42,20	Diagnosis by two rheumatologists using ACR criteria	NHS:n=76 597(624), NHSII:n=91 393(383), validated semi-quantitative FFQ	red/processed meat, sugar-sweetened beverages,trans fat, sodium,nuts, vegetables, fruits, PUFA, alcohol, whole grain, long-chain n-3 PUFA	1, 4, 5,15, 16, 17, 20, 21, 43

Karlson 2003(NHS)[13]	US,female,34-59 , 8	Diagnosis by two rheumatologists using ACR criteria	n=83 124(731), validated semi-quantitative FFQ	caffeinated coffee, decaffeinated coffee, total coffee, tea,caffeine	1, 4, 5, 14, 15, 16, 17, 28, 35, 43
Krok-Schoen 2018(WHI-OS)[14]	US,female,55-79 ,8	Self-reported	n=80 551(3 348), validated semi-quantitative FFQ	alcohol, fruits,vegetables, red meat, long-chain n-3 PUFA, n-6 fatty acids	1, 3, 4, 6, 21, 25, 29, 46
Lamichhane 2019(WHI-OS)[15]	US,female,55-79 ,8	Self-reported & DMARD use	n=76 853(185), validated semi-quantitative FFQ	total coffee, caffeinated coffee, decaffeinated coffee, unfiltered coffee, filtered coffee, tea, caffeine	1,3, 4,5, 6, 9, 14, 16
Lu 2014(NHS & NHSII)[16]	NHS:US,female, 30-55,28 NHSII:US,female,25-42,20	Diagnosis by two rheumatologists using ACR criteria	NHS:n=82 472(580), NHSII:n=110 737(323), validated semi-quantitative FFQ	alcohol, beer,wine,liquor	1, 4, 5, 12, 13, 15, 16, 17, 19, 21, 43
Mikuls 2002(IWHS)[17]	US,female,55-69 ,11	Diagnosis by two rheumatologists using ACR criteria	n=31 336(158), validated semi-quantitative FFQ	total coffee, caffeinated coffee, decaffeinated coffee,tea,caffeine	1, 5, 9, 14, 15, 16
Pedersen 2005(DCH cohort)[18]	Denmark, both sexes,50-64,5.3	Diagnosis by rheumatologists using ACR criteria	n=56 691(69), validated self-administered FFQ	fish, lean fish ,medium fat fish,fat fish,olive oil,all fruits and fruit juices ,citrus fruits,all vegetables/vegetable juices, all vegetables/fruits/juices, b-carotene, vitamin A, vitamin E, vitamin C, vitamin D, zinc, selenium, iron, red meat/fish/poultry/processed meat, red meat, coffee	1, 2, 5, 6
Rubin 2020(DCH cohort)[19]	Denmark, both sexes,50-64,5.3	Diagnosis by rheumatologists using ACR criteria	n=56 075(797), validated self-administered FFQ	dietary fibre, meat	1, 2, 5, 6, 11, 14, 21, 27

Sparks 2019(NHS & NHSII)[20]	NHS:US,female, 30-55,20 NHSII:US,female,25-42,24	Diagnosis by rheumatologists using ACR criteria	n=166 013(1 080), validated self-administered FFQ	fish, marine n-3 PUFA	1, 4, 5, 14, 46, 20, 21, 37, 39, 40, 41, 42, 45, 46
VanEvery 2021(Kailuan Study)[21]	China, both sexes, mean age:51.8, 10	Diagnosis by two rheumatologists using ACR criteria	n=87 118(87), validated self-reported alcohol consumption questionnaire	alcohol	1, 2, 4, 5
Cerhan 2002(IWHS)[22]	US,female,55-69,11	Diagnosis by two rheumatologists using ACR criteria	n=31 336(158), validated semi-quantitative FFQ	alcohol, beer, red wine, white wine, liquor	1
Benito-Garcia 2007(NHS)[23]	US,female,30-55, 22	Diagnosis by two rheumatologists using ACR criteria	n=82 063(546), validated semi-quantitative FFQ	total protein, animal protein, vegetable protein,total iron intake (diet and supplements),dietary iron,iron from supplements,heme iron intake,meat,red meat,poultry meat,fish	1, 4, 5, 43
Hu 2014(NHS & NHSII)[24]	NHS:US,female, 30-55,28 NHSII:US,female,25-42,20	Diagnosis by two rheumatologists using ACR criteria	NHS:n=79 570(559), NHSII:n=107 330(298), validated semi-quantitative FFQ	sugar-sweetened soda, diet soda	1, 4, 5, 12, 13, 14, 16, 17, 18, 21, 26, 27,43
Nguyen 2022(E3N-EPIC)[25]	France,female, 52.5±6.5,10	Self-reported & diagnosis by two rheumatologists using ACR criteria	n=62 629(480), validated French Dietary History Questionnaire	total fish, lean fish, oily fish	1, 4, 5, 6, 11, 12, 21
Heliövaara 2007(Mobile Clinic Health Examination	Finland,both sexes,20-98,13	Social Insurance Institution's population register	n=18 981(126), validated self-filled questionnaires	coffee, alcohol	1, 2

Survey)[26]					
Mazzucca 2022(UK Biobank)[27]	UK,both sexes,40-69,12	ICD-10	n=479 494(2 819),validated semi-quantitative FFQ	cooked vegetables, fruits,cheese,oily fish,processed meat,beef,lamb,pork,alcohol, tea,coffee,decaffeinated coffee, ground coffee, instant coffee,other coffee	1, 2, 4, 5, 6, 12, 13, 14, 27
Eun 2020(NHIS)[28]	South Korea,female,61.4,9	N/A	n=1 357 736(6 056), self-report questionnaires	alcohol	1, 4, 5, 12, 13, 14, 16, 17, 26, 27, 28, 43
Ro 2022 (NHIS-NSC)[29]	South Korea,both sexes,40-79,14	ACR criteria, positive for either rheumatoid factor or anti-citrullinated peptide antibod	n=517 053(1 948), self-report questionnaires	alcohol	1, 4, 5, 12, 13, 14, 27
Ascione 2023(E3N-EPIC Cohort Study)[30]	France, female, 52.5±6.5, 11.7	1987 ACR criteria	n=62 149(481), validated FFQ	total alcohol, beer, wine, liquor, total coffee, caffeinated coffee, decaffeinated coffee, tea	1, 4, 5, 6, 11, 12, 21

IWHS= Iowa Women's Health Study; NHS & NHSII=Nurses' Health Study and Nurses' Health Study II Cohorts; SMC= Swedish Mammography Cohort; SNMC= Swedish National March Cohort; HS-FFQ = high school-food frequency questionnaire; EPIC=European Prospective Investigation into Cancer study; E3N-EPIC= Etude Epidémiologique auprès des femmes de la Mutuelle générale de l' Education Nationale; WHI-OS= Women's Health Initiative Observational Study and Clinical Trials cohort; DCH = Diet, Cancer, and Health cohort; FFQ= food frequency questionnaire; F = female, SIICD = The Social Insurance Institution's Coronary Heart Disease Study; UKB = UK Biobank; ACR = the American College of Rheumatology; ICD-10 = International Classification of Diseases, 10th Revision; aMed: HLIS= Healthy Lifestyle Index Score; BMI=body mass index; PUFA= polyunsaturated fatty acid; trans-FA= trans fatty acid; SFA=saturated fatty acid; MFA: monounsaturated fatty acids; Adjustment: age(1), sex(2), race/ethnicity(3), BMI(4), smoking status(5), education(6), civil status(7), occupation status(8), marital status(9), sugar-sweetened soda(9), total energy intake(10), gastrointestinal transit(11), physical activity(12), family income(13), alcohol use(14), age at menopause(15), use of hormone replacement therapy(16), parity(17), multivitamin use(18), beverages(19), cohort(20), total energy(21), use of aspirin (22), dairy consumption(23), fish consumption(24), fruits(25), age at menarche(26), co-morbidity(e.g., diabetes mellitus, cancer, cardiovascular disease)(27), oral contraceptive use(28), memory loss(29), decaffeinated coffee consumption(30), Socioeconomic Status(31), sunscreen use and sun sensitivity(32), birth weight(33), latitude of residence(34); age at first birth(35), tea consumption(36), α -linolenic acid(37), coffee consumption(38), trans fatty acids (g/d)(39), saturated fatty acids (g/d)(40), omega-6 polyunsaturated fatty acids (g/d)(41), protein (g/d)(42), breastfeeding(43), β -cryptoxanthin intake(44), monounsaturated fatty acids (g/d)(45); questionnaire circle(46); meat(47)

Table S6. Characteristics of 14 included cohort studies

**Cohort(country)**	**N of studies**	**Follow-up**	**Gender**	**Dietary assessment method and frequency**	**Dietary exposure**
IWHS(US)	4	1987-1997	Female	FFQ; Single at baseline	vitamins, carotenoids, minerals, fruits, legumes, vegetables, milk, butter, coffee, tea, caffeine, alcohol
NHS(US)	11	1980-2002	Female	FFQ; Baseline and repeated every 4 years	vitamins, carotenoids,Mediterranean diet, red/processed meat, fish, vegetables, whole grain, nuts, MUFA:SFA ratio, beverages, trans fat, n-3 PUFA, coffee, tea, caffeine, alcohol, Empirical Dietary Inflammatory Pattern, fish, total protein, animal protein, vegetable protein, total meat
NHSII(US)	9	1991-2001	Female	FFQ; Baseline and repeated every 4 years	vitamins, carotenoids,Mediterranean diet, red/processed meat, fish, vegetables, whole

					grain, nuts, MUFA:SFA ratio, beverages, trans fat, n-3 PUFA, coffee, tea, caffeine, alcohol, Empirical Dietary Inflammatory Pattern, fish, total protein, animal protein, vegetable protein, total meat
SMC(Sweden)	3	2003-2014	Female	FFQ, Repeated in 1987 and 1997	alcohol, meat, milk, cheese, fish, n-3 PUFA
SNMC(Sweden)	1	1997-2016	Both sexes	Questionnaire for epidemiological research, Single at baseline	alcohol
EPIC-Nortfolk(UK)	1	1997-2010	Both sexes	FFQ, Single at baseline	alcohol
E3N-EPIC(France)	3	2004-2014	Female	FFQ, Single at baseline	vegetables, legumes, fruits, cereal products, fish, meat, dairy product, unsaturated fat, alcohol, Mediterranean diet, total coffee, caffeinated coffee, decaffeinated coffee, tea, total alcohol, beer, wine, liquor
WHI-OS(US)	2	8yrs	Female	FFQ; Single at baseline	alcohol, fruits,vegetables, red meat, energy, n-3 fatty

					acids, n-6 fatty acids, coffee, tea, caffeine
DCH(Denmark)	2	1997-2018	Both sexes	FFQ, Single at baseline	fish, olive oil, fruits, vegetables, vitamins, minerals, red meat, coffee, dietary fibre, meat
Kailuan(China)	1	2006-2018	Both sexes	Questionnaire, Single at baseline	alcohol
UK Biobank(UK)	1	2005-2017	Both sexes	FFQ, Single at baseline	vegetables,oily fish,fresh fruit, processed meat, poultry, beef, lamb, pork, cheese, bread, breakfast cereal, coffee, tea
NHIS-NSC(South Korea)	1	2002-2016	Both sexes	self-report questionnaires, Single at baseline	alcohol
NHIS(South Korea)	1	2009-2018	Female	self-report questionnaires; Single at baseline	alcohol
MCHES(Finland)	1	1977-1989	Both sexes	Mobile Clinic Health Examination Survey, Single at baseline	coffee, alcohol

*IWHS= Iowa Women's Health Study; NHS & NHSII=Nurses' Health Study and Nurses' Health Study II Cohorts; SMC= Swedish Mammography Cohort; SNMC= Swedish National March Cohort; EPIC=European Prospective Investigation into Cancer study;DCH = Diet, Cancer, and Health cohort; FFQ= food frequency questionnaire;WHI-OS= Women's Health Initiative Observational Study and Clinical Trials cohort; MCHES=Mobile Clinic Health Examination Survey; NHIS-NSC=Korean National Health Insurance Service (NHIS)-national sample cohort

Table S8. Scoring for the food groups, beverages and food components of NutriGrade for the risk of rheumatoid arthritis

Exposure	Outcome	Risk of bias[1]	Precision[2]	Heterogeneity[3]	Directness[4]	Publication bias[5]	Funding bias[6]	Effect size[7]	Dose-response[8]	Sum	NutriGrade
Total alcohol	RA	1.5	1	0	0	0.5	1	1	1	6	Moderate
Total alcohol	Seropositive RA	1.5	1	1	1	0.5	1	1	1	7	Moderate
Wine	RA	1.5	0	1	1	0	1	0	0	4.5	Low
Beer	RA	1.5	0	1	1	0	1	1	1	6.5	Very low
Liquor	RA	1.5	0	1	1	0	1	0	0	4.5	Low
Total coffee	RA	1.5	0	0	0	0	1	0	1	3.5	Very low
Caffeinated coffee	RA	1.5	0	1	0	0	1	1	0	4.5	Low
Decaffeinated coffee	RA	1.5	0	0	0	0.5	1	0	0	3	Very low
Decaffeinated coffee	Rheumatoid Factor-positive RA	1.5	0	0	1	0	1	0	0	3.5	Very low
Caffeine	RA	1.5	0	0	0	0	1	0	0	2.5	Very low
Tea	RA	1.5	0	1	0	0.5	1	1	1	6	Moderate

Tea	Rheumatoid Factor-positive RA	1.5	0	0	1	0	1	0	0	3.5	Very low
Sugar-sweetened soda	RA	2	0	0	1	0	1	0	0	4	Low
Fruit	RA	1.5	1	0	1	0.5	1	1	1	7	Moderate
Vegetable	RA	1.5	0	0	1	0.5	1	0	1	5	Low
Total meat	RA	2	0	1	1	0	1	0	0	5	Low
Red meat	RA	1.5	0	1	0	0	1	0	0	3.5	Very low
Processed meat	RA	1.5	0	1	1	0	1	0	0	4.5	Low
Poultry	RA	1.5	0	1	1	0	1	0	0	4.5	Low
Total fish	RA	2	0	1	1	0	1	0	0	5	Low
Oily fish	RA	1.5	0	1	1	0	1	0	1	5.5	Low
Legumes	RA	2	0	1	1	0	1	0	0	5	Low
Total dairy	RA	2	0	0	1	0	1	0	0	4	Low
Milk	RA	1.5	0	1	1	0	1	0	0	4.5	Low

Cheese	RA	1.5	0	0	1	0	1	0	0	3.5	Very low
Cereal products	RA	2	0	1	1	0	1	1	1	7	Moderate
Vitamin C	RA	1.5	0	1	1	0	1	0	0	4.5	Low
Vitamin D	RA	2	0	1	1	0	1	0	0	5	Low
Vitamin D supplement	RA	2	0	0	1	0	1	0	1	5	Low
Vitamin A	RA	1.5	0	1	1	0	1	0	0	4.5	Low
Vitamin E	RA	1.5	0	1	1	0	1	0	0	4.5	Low
α-carotenoid	RA	1.5	0	0	1	0	1	0	0	3.5	Very low
β -carotenoid	RA	1.5	0	0	1	0	1	0	0	3.5	Very low
β -cryptoxanthin	RA	1.5	0	1	1	0	1	0	0	4.5	Low
Lycopene	RA	1.5	0	1	1	0	1	0	0	4.5	Low
Lutein/zeaxanthin	RA	1.5	0	0	1	0	1	0	0	3.5	Very low
N-3 PUFA	RA	1.5	0	0	0	0	1	0	0	2.5	Very low

[1] Modifications of original NutriGrade-scoring system: Study quality Newcastle Ottawa Scale: ≥8 (2 points); ≥7-<8 (1.5 points), ≥6-<7(1 point).

[2] >5000 cases and the 95%CI excludes the null value (1 point), <5000 cases or >5000 cases but 95%CI includes the null value (0 point).

[3] 0 to 1 point on the basis of I^2.

[4] No important differences in the population or hard clinical outcomes (1 point), important differences in the population (0 point).

[5] <5 studies or sever evidence of bias or publication bias not assessed (0 point), no evidence of bias for 5-9 studies or moderate evidence of bias for ≥10 studies (0.5 point), no evidence of bias for ≥10 studies (1 point).

[6] Funded by academic institutions or research institutions (1 point), funded by private institutions, foundations, or nongovernmental organizations (0.5 point), Industry funding or conflict of interest (0 point).

[7] No effect (RR: 0.80-1.20) when comparing the highest vs. lowest category (0 point), moderate effect size (RR: <0.80-0.50 and >1.20-2, and corresponding test is statistically significant) when comparing the highest vs. lowest category (1 point), large effect size (RR: <0.50 and >2.00, and corresponding test is statistically significant) when comparing the highest vs. lowest category (2 point).

[8] No dose-response analysis or dose-response analysis with corresponding statistical test no significant (0 point), significant linear or nonlinear dose-response relationship (1 point)

Table S9. Newcastle Ottawa Scale scores of the included prospective cohort studies

Study (Supplemental reference)	Selection				Comparability	Exposure			Total score
	Exposed cohort[1]	Non-exposed cohort[2]	Exposure assessment[3]	Demonstration[4]	Confounding[5]	Outcome assessment[6]	Follow-up duration[7]	Non-response rate[8]	
Cerhan et al. 2003	1	1	1	1	2	1	0	1	8
Costenbader et al. 2008	1	1	1	1	1	1	1	0	7
Costenbader et al. 2010	1	1	1	1	1	1	1	0	7
Di Giuseppe et al. 2012	1	1	1	1	0	1	1	0	6
Sundström et al. 2019	1	1	1	1	2	1	1	0	7
Di Giuseppe et al. 2013	1	1	1	1	2	1	1	0	8
Hedenstierna et al. 2021	1	1	0	1	2	1	1	0	7
Hiraki et al. 2012	1	1	1	1	2	1	1	1	9
Merlino et al. 2004	1	1	1	1	2	1	1	0	8
Lahiri et al. 2012	1	1	1	1	2	1	0	1	7
Nguyen et al. 2020	1	1	1	1	2	1	1	1	9
Hu et al. 2015	1	1	1	1	2	1	1	0	8
Hu et al. 2017	1	1	1	1	2	1	1	0	8
Karlson et al. 2003	1	1	1	1	1	1	1	0	7
Krok-Schoen et al. 2018	0	1	1	1	2	1	0	0	6
Lamichhane et al. 2019	0	1	1	1	2	1	1	0	7
Lu et al. 2014	1	1	1	1	2	1	1	1	9
Sparks et al. 2019	1	1	1	1	2	1	1	1	9
Mikuls et al. 2002	1	1	1	1	1	1	1	1	8
Pedersen et al. 2005	1	1	1	1	1	1	0	0	6
Rubin et al. 2020	1	1	1	1	2	1	1	0	8
Sparks et al. 2019	1	1	1	1	2	1	1	0	8
VanEvery et al. 2021	1	1	0	1	1	1	1	0	6

Cerhan et al. 2002	1	1	1	1	0	1	1	0	6
Benito-Garcia et al. 2007	1	1	1	1	2	1	1	0	8
Hu et al. 2014	1	1	1	1	2	1	1	0	8
Nguyen et al. 2022	1	1	1	1	2	1	1	0	8
Heliövaara et al. 2000	1	1	1	1	0	1	1	0	6
Mazzucca et al. 2022	1	1	1	1	2	1	0	0	7
Eun et al. 2020	0	1	0	1	1	1	0	0	5
Ro et al. 2022	1	1	0	1	1	1	1	0	6
Ascione et al. 2023	1	1	1	1	2	1	1	1	9

[1]For the representativeness of the exposed cohort, if the participants were derived from general population, then this item would be given 1 point; if the participants were derived from occupational population, disease population, or population with higher risk factor (smoking population), then this item would be given 0 point. [2]For the selection of the non-exposed cohort, if drawn from the same original population as the exposed cohort, then this item would be given 1 point; otherwise 0 point. [3]For the ascertainment of exposure, if intakes of food and nutrient were assessed by structured dietary assessment tools (e.g., 24h-recall, and FFQ), then this item would be given 1 point; if not, this item would be given 0 point. [4]For the demonstration that RA was not present at start of study, this item would be given 1 point; otherwise 0 points. [5]For comparability of cohorts, if the model adjusted for age, sex, and another socioeconomic factor, then this item would be given 1 point; if the model adjusted for smoking status, alcohol intake, physical activity and other dietary information, then this study would be given additional 1 point. [6]For the assessment of outcome, if the outcome was identified through medical records, or registered data, then this item would be given 1 point; otherwise this item would be given 0 point. [7]For follow-up duration, if it was 10 years or above, then this item would be given 1point; otherwise 0 point. [8]For the adequacy of follow up of cohorts, if the rate of loss to follow-up was less than 10%, then this item would be given 1 point; if ≥10% or no information on the rate of loss to follow-up, then 0 point.

Table S10(a). Subgroup analyses for total alcohol consumption and risk of rheumatoid arthritis

	n	RR	95% CI	I^2 (%)	Q within[1]	Q between[2]	P[3]
All studies	8	0.76	[0.68,0.85]	56	15.57		
Gender							
Both sexes	3	0.72	[0.65,0.80]	82	10.91	2.22	0.14
Female	5	0.80	[0.74,0.87]	0	2.44		
Geographic location							
Europe	4	0.72	[0.66, 0.80]	56	6.80	8.27	0.02
America	3	0.80	[0.73, 0.88]	0	0.50		
Asia	1	1.98	[0.93, 4.22]	-	0		
Follow-up duration							
<10 years	3	0.77	[0.72, 0.83]	15	2.35	0.12	0.73
≥10 years	5	0.75	[0.65, 0.87]	70	13.10		
Number of cases							
<500	4	0.86	[0.70,1.06]	57	6.97	1.33	0.25
≥500	4	0.76	[0.71,0.81]	59	7.27		
Outcome assessment							
Including self-reported data	4	0.81	[0.74,0.88]	0	1.07	3.18	0.07
Register data	4	0.72	[0.65,0.79]	74	11.31		

[1]*Q* within each subgroup;

[2]*Q* between subgroups;

[3]*P* values for comparisons between subgroups.

Table S10 (b) Subgroup analyses for total alcohol consumption and risk of seropositive rheumatoid arthritis

	n	RR	95% CI	I^2 (%)	Q_{within}[1]	$Q_{between}$[2]	P[3]
All cohorts	5	0.84	[0.73,0.96]	0	3.30		
Gender							
Both sexes	1	0.75	[0.61,0.92]	0	1.39	1.92	0.17
Female	4	0.91	[0.76,1.07]	-	0		
Geographic location							
Europe	1	0.66	[0.41, 1.07]	-	0	1.32	0.51
America	2	0.81	[0.61, 1.07]	0	0.80		
Asia	2	0.88	[0.75, 1.02]	15.8	1.19		
Number of cases							
<500	3	0.77	[0.60,0.98]	0	1.31	0.80	0.37
≥500	2	0.87	[0.75,1.02]	15.8	1.19		

[1]*Q* within each subgroup;

[2]*Q* between subgroups;

[3]*P* values for comparisons between subgroups.

Table S10 (c). Subgroup analyses for total coffee consumption and risk of rheumatoid arthritis

	n	RR	95% CI	I^2 (%)	Q_{within} [1]	$Q_{between}$ [2]	P[3]
All studies	6	1.16	[0.94,1.42]	63	13.52		
Age (mean/median)							
<50 years	2	1.73	[0.59,5.07]	57.4	2.35	0.59	0.44
≥50 years	4	1.12	[0.88,1.43]	64.1	8.35		
Gender							
Both sexes	2	0.92	[0.83,1.00]	73.1	3.72	0.09	0.76
Female	4	1.26	[1.05,1.51]	0	0.50		
Geographic location							
America	3	1.27	[1.00,1.61]	0	0.49	0.33	0.56
Europe	3	0.92	[0.83,1.00]	74.5	7.83		
Follow-up duration							
<10 years	2	1.01	[0.74,1.39]	58.9	2.43	1.43	0.23
≥10 years	4	1.31	[0.98,1.74]	0	2.74		
N of cases							
<500	5	1.28	[1.07,1.53]	0	2.74	10.78	<0.01
≥500	1	0.91	[0.83,1.00]	-	0		
Outcome assessment							
Including self-reported data	4	1.26	[1.05,1.51]	0	0.50	9.30	<0.01
Register data	2	1.56	[0.39,6.23]	73.1	3.72		
Dietary assessment tool							
FFQ	5	0.97	[0.89,1.06]	60.5	10.14	3.39	0.06
Not mentioned	1	3.95	[0.89,17.52]	-	0		

[1] *Q* within each subgroup;

[2] *Q* between subgroups;

[3] *P* values for comparisons between subgroups.

Table S10 (d). Subgroup analyses for decaffeinated coffee consumption and risk of rheumatoid arthritis

	**n**	**RR**	**95% CI**	I^2 **(%)**	Q_{within}[1]	$Q_{between}$[2]	P[3]
All studies	5	1.34	[0.95,1.88]	66	11.90		
Age (mean/median)							
<50 years	1	1.00	[0.59,1.68]	-	0	0.83	0.36
≥50 years	4	1.29	[1.08,1.54]	72.9	11.08		
Gender							
Both sexes	1	0.96	[0.68,1.35]	-	0	3.11	0.08
Female	4	1.37	[1.13,1.65]	65.9	8.79		
Geographic location							
America	3	1.66	[1.22,2.26]	68	6.25	4.48	0.03
Europe	2	1.12	[0.92,1.37]	14.5	1.17		
Follow-up duration							
<10 years	2	1.10	[0.81,1.48]	62	2.63	1.13	0.29
≥10 years	3	1.33	[1.09,1.63]	75	8.15		
N of cases							
<500	4	1.36	[1.13,1.65]	65.9	8,79	3.11	0.08
≥500	1	0.96	[0.68,1.35]	-	0		
Outcome assessment							
Including self-reported data	4	1.37	[1.13,1.65]	65.9	8.79	3.11	0.08
Register data	1	0.96	[0.68,1.35]	-	0		

[1]*Q* within each subgroup;

[2]*Q* between subgroups;

[3]*P* values for comparisons between subgroups.

Table S10 (e). Subgroup analyses for tea consumption and risk of rheumatoid arthritis

	n	RR	95% CI	I^2 (%)	Q_{within}[1]	$Q_{between}$[2]	P[3]
All studies	5	0.88	[0.79,0.97]	50	8.01		
Age (mean/median)							
<50 years	1	1.10	[0.73,1.66]	-	0	0.16	0.69
≥50 years	4	1.20	[1.10,1.31]	64.0	8.33		
Gender							
Both sexes	1	1.23	[1.12,1.36]	-	0	2.00	0.16
Female	4	1.04	[0.85,1.28]	53.8	6.49		
Geographic location							
America	3	1.06	[0.75,1.49]	69.1	6.47	0.49	0.48
Europe	2	1.20	[1.10,1.32]	34.5	1.53		
Follow-up duration							
<10 years	3	1.00	[0.80,1.24]	55	4.44	3.16	0.08
≥10 years	2	1.24	[1.12,1.36]	0	0.89		
N of cases							
<500	4	1.04	[0.85,1.28]	53.8	6.49	2.00	0.16
≥500	1	1.23	[1.11,1.36]	-	0		
Outcome assessment							
Including self-reported data	4	1.04	[0.85,1.28]	53.8	6.49	2.00	0.16
Register data	1	1.23	[1.12,1.36]	-	0		

[1]Q within each subgroup;

[2]Q between subgroups;

[3]P values for comparisons between subgroups.

Table S10 (f). Subgroup analyses for fruit consumption and risk of rheumatoid arthritis

	n	RR	95% CI	I^2 (%)	Q_{within}[1]	$Q_{between}$[2]	P[3]
All studies	5	0.88	[0.79,0.97]	50	8.01		
Age (mean/median)							
<50 years	1	1.03	[0.78,1.37]	-	0	1.51	0.22
≥50 years	4	0.86	[0.81,0.91]	54	6.50		
Gender							
Both sexes	1	0.92	[0.84,1.01]	-	0	3.01	0.08
Female	4	0.82	[0.76,0.90]	40	4.99		
Geographic location							
America	3	0.81	[0.74,0.88]	39	3.27	4.67	0.03
Europe	2	0.92	[0.85,1.00]	0	0.07		
Follow-up duration							
<10 years	3	0.85	[0.80,0.91]	65	5.67	2.15	0.14
≥10 years	2	0.98	[0.82,1.17]	0	0.19		
N of cases							
<500	3	0.94	[0.80,1.11]	0	1.78	1.11	0.29
≥500	2	0.85	[0.80,0.91]	80	5.11		
Outcome assessment							
Including self-reported data	4	0.82	[0.76,0.90]	40	5.00	3.01	0.08
Register data	1	0.92	[0.84,1.01]	0	0		

[1]Q within each subgroup;

[2]Q between subgroups;

[3]P values for comparisons between subgroups.

Table S10 (g). Subgroup analyses for vegetables consumption and risk of rheumatoid arthritis

	n	RR	95% CI	I^2 (%)	Q_{within}[1]	$Q_{between}$[2]	P[3]
All studies	5	0.90	[0.76,1.06]	81	20.88		
Age (mean/median)							
<50 years	1	0.84	[0.63,1.12]	-	0	0.2	0.65
≥50 years	4	0.90	[0.84,0.97]	86	20.68		
Gender							
Both sexes	1	1.13	[0.99,1.28]	-	0	18.26	<0.001
Female	4	0.81	[0.74,0.88]	0	2.62		
Geographic location							
America	3	0.78	[0.71,086]	0	0.30	18.9	<0.001
Europe	2	1.08	[0.97,1.21]	41	1.68		
Follow-up duration							
<10 years	3	0.89	[0.83,0.97]	90	20.43	0.02	0.89
≥10 years	2	0.91	[0.76,1.08]	0	0.43		
N of cases							
<500	3	0.88	[0.75,1.04]	0	1.15	0.06	0.81
≥500	2	0.89	[0.73,0.97]	95	19.68		
Outcome assessment							
Including self-reported data	4	0.81	[0.74,0.88]	0	2.62	18.26	<0.001
Register data	1	1.13	[0.99,1.28]	-	0		

[1]*Q* within each subgroup;

[2]*Q* between subgroups;

[3]*P* values for comparisons between subgroups.

Table S11 (a). Sensitivity analyses for food group consumption and risk of rheumatoid arthritis: excluding one study at a time[1]

Study (Supplemental reference)	Estimate	95% Confidence Interval	
Total alcohol			
Included all studies	0.76	0.68	0.85
Omitting Di Giuseppe 2012	0.77	0.68	0.87
Omitting Hedenstierna 2021	0.79	0.73	0.84
Omitting Ascione 2023	0.74	0.66	0.84
Omitting Krok-Schoen 2018	0.75	0.64	0.88
Omitting Hu 2017	0.77	0.66	0.89
Omitting VanEvery 2021	0.75	0.68	0.83
Omitting Cerhan 2002	0.76	0.67	0.86
Omitting Mazzucca 2022	0.77	0.65	0.92
Total alcohol(seropositve RA)			
Included all studies	0.84	0.73	0.96
Omitting Lu 2014 (NHS)	0.80	0.65	0.98
Omitting Lu 2014(NHSII)	0.86	0.75	0.98
Omitting Eun 2020	0.86	0.75	0.99
Omitting Ro 2022	0.75	0.61	0.93
Omitting Ascione 2023	0.86	0.75	0.99
Wine			
Included all studies	0.95	0.79	1.14
Omitting Di Giuseppe 2012	0.96	0.78	1.18
Omitting Lu 2014(NHS)	0.86	0.70	1.06
Omitting Lu 2014(NHSII)	0.98	0.81	1.18
Omitting Cerhan 2002	0.95	0.77	1.17
Omitting Ascione 2023	0.97	0.74	1.28
Beer			
Included all studies	0.87	0.74	1.02
Omitting Di Giuseppe 2012	0.87	0.73	1.03
Omitting Lu 2014(NHS)	0.90	0.76	1.06
Omitting Lu 2014(NHSII)	0.86	0.73	1.02
Omitting Cerhan 2002	0.86	0.73	1.02
Omitting Ascione 2023	0.78	0.59	1.03
Liquor			
Included all studies	0.93	0.77	1.11
Omitting Di Giuseppe 2012	0.93	0.78	1.13
Omitting Lu 2014(NHS)	0.90	0.73	1.13
Omitting Lu 2014(NHSII)	0.93	0.78	1.12
Omitting Cerhan 2002	0.91	0.74	1.10
Omitting Ascione 2023	0.95	0.75	1.20
Total coffee			
Included all studies	1.16	0.94	1.42
Omitting Lamichhane 2019	1.15	0.91	1.45

Omitting Karlson 2003	1.17	0.90	1.52
Omitting Mikuls 2002	1.12	0.91	1.39
Omitting Mazzucca 2022	1.12	0.92	1.37
Omitting Heliovaara 2000	1.10	0.87	1.40
Omitting Ascione 2023	1.16	0.89	1.50
Caffeinated coffee			
Included all studies	1.30	1.09	1.54
Omitting Mikuls 2002	1.35	1.12	1.62
Omitting Lamichhane 2019	1.29	1.07	1.55
Omitting Karlson 2003	1.25	1.00	1.54
Omitting Ascione 2023	1.29	1.03	1.62
Decaffeinated coffee			
Included all studies	1.34	0.95	1.88
Omitting Mazzucca 2022	1.48	1.00	2.18
Omitting Lamichhane 2019	1.28	0.86	1.90
Omitting Karlson 2003	1.43	0.95	2.15
Omitting Mikuls 2002	1.14	0.96	1.37
Omitting Ascione 2023	1.40	0.88	2.22
Decaffeinated coffee(RF-positive RA)			
Included all studies	1.80	0.90	3.58
Omitting Karlson 2003	2.64	1.46	4.78
Omitting Mikuls 2002	1.30	0.85	1.99
Caffeine			
Included all studies	1.24	0.93	1.64
Omitting Karlson 2003	1.30	0.68	2.49
Omitting Lamichhane 2019	1.13	0.88	1.44
Omitting Mikuls 2002	1.39	0.94	2.06
Tea			
Included all studies	1.15	1.00	1.33
Omitting Mikuls 2002	1.20	1.10	1.32
Omitting Lamichhane 2019	1.11	0.94	1.33
Omitting Karlson 2003	1.13	0.92	1.39
Omitting Mazzucca 2022	1.04	0.85	1.28
Omitting Ascione 2023	1.22	1.11	1.34
Tea(RF-positive RA)			
Included all studies	0.64	0.15	2.80
Omitting Karlson 2003	0.26	0.06	1.11
Omitting Mikuls 2002	1.20	0.61	2.35
Sugar-sweetened soda			
Included all studies	1.13	0.80	1.58
Omitting Hu 2014(NHS)	0.94	0.75	1.18
Omitting Hu 2014(NHSII)	1.19	0.71	2.00
Omitting Ascione 2023	1.29	0.82	2.01
Fruit			

Included all studies	0.88	0.79	0.97
Omitting Cerhan 2013	0.89	0.79	0.99
Omitting Nguyen 2020	0.87	0.76	0.96
Omitting Hu 2017(NHS,NHSII)	0.86	0.76	0.96
Omitting Krok-Schoen 2018	0.92	0.85	1.00
Omitting Mazzucca 2022	0.86	0.74	0.99
Vegetable			
Included all studies	0.90	0.76	1.06
Omitting Cerhan 2013	0.92	0.77	1.10
Omitting Nguyen 2020	0.88	0.72	1.09
Omitting Hu 2017(NHS,NHSII)	0.91	0.74	1.11
Omitting Krok-Schoen 2018	0.96	0.80	1.15
Omitting Mazzucca 2022	0.81	0.73	0.91
Legumes			
Included all studies	0.97	0.80	1.19
Omitting Cerhan 2013	0.98	0.79	1.22
Omitting Nguyen 2020	0.95	0.61	1.48
Total meat			
Included all studies	0.94	0.82	1.06
Omitting Sundstrom 2019	0.91	0.80	1.05
Omitting Nguyen 2020	0.90	0.77	1.04
Omitting Rubin 2020	1.00	0.85	1.18
Omitting Benito-Garcia 2007	0.95	0.90	1.12
Red meat			
Included all studies	1.06	0.91	1.24
Omitting Krok-Schoen 2018	0.95	0.76	1.19
Omitting Benito-Garcia 2007	1.13	1.03	1.24
Omitting Sundstrom 2019	1.02	0.79	1.32
Processed meat			
Included all studies	1.00	0.88	1.13
Omitting Mazzucca 2022	0.84	0.58	1.21
Omitting Sundstrom 2019	1.02	0.89	1.17
Poultry			
Included all studies	1.11	0.95	1.28
Omitting Sundstrom 2019	1.15	0.98	1.35
Omitting Mazzucca 2022	1.05	0.79	1.38
Omitting Benito-Garcia 2007	1.06	0.85	1.33
Total fish			
Included all studies	0.92	0.78	1.08
Omitting Di Giuseppe 2013	0.97	0.81	1.16
Omitting Nguyen 2020	0.83	0.64	1.08
Omitting Sparks 2019	0.87	0.64	1.20
Oily fish			
Included all studies	0.91	0.78	1.06

Omitting Mazzucca 2022	1.01	0.81	1.25
Omitting Nguyen 2020	0.86	0.75	0.99
Milk			
Included all studies	1.06	0.84	1.34
Omitting Sundstrom 2019	1.04	0.69	1.57
Omitting Merlino 2004	1.07	0.80	1.43
Cheese			
Included all studies	0.94	0.64	1.39
Omitting Mazzucca 2022	1.20	0.81	1.77
Omitting Sundstrom 2019	0.80	0.69	0.93
Total dairy			
Included all studies	0.97	0.72	1.30
Omitting Nguyen 2020	0.86	0.53	1.41
Omitting Merlino 2004	1.11	0.92	1.34
Omitting Sundstrom 2019	0.89	0.53	1.49
Cereal product			
Included all studies	0.87	0.80	0.96
Omitting Nguyen 2020	0.87	0.79	0.95
Omitting Mazzucca 2022	0.89	0.73	1.07
Omitting Hu 2017(NHS, NHSII)	0.88	0.80	0.97

[1]Effect sizes were pooled using random-effects model

Table S11 (b) Sensitivity analyses for nutrient intake and risk of rheumatoid arthritis: excluding one study at a time[1]

Study (Supplemental reference)	Estimate	95% Confidence Interval	
N-3 PUFA			
Included all studies	0.90	0.73	1.10
Omitting Di Giuseppe et al	0.98	0.86	1.11
Omitting Hu et al(NHS,NHSII)	0.87	0.59	1.28
Omitting Krok-Schoen et al(WHI)	0.80	0.64	1.01
Vitamin C			
Included all studies	0.98	0.76	1.27
Omitting Cerhan et al(IWHS)	1.10	0.89	1.36
Omitting Costenbader et al(NHS)	0.88	0.58	1.34
Omitting Costenbader et al(NHSII)	0.92	0.61	1.39
Vitamin D			
Included all studies	0.79	0.62	1.00
Omitting Hiraki et al(NHS)	0.80	0.52	1.24
Omitting Hiraki et al(NHSII)	0.74	0.57	0.97
Omitting Merlino et al(IWHS)	0.86	0.64	1.16
Vitamin D supplement			
Included all studies	1.08	0.55	2.10
Omitting Costenbader et al(NHS)	1.18	0.35	4.00
Omitting Costenbader et al(NHSII)	0.80	0.54	1.21
Omitting Merlino et al(IWHS)	1.43	0.64	3.21
Vitamin A			
Included all studies	1.04	0.82	1.32
Omitting Costenbader et al(NHS)	0.90	0.57	1.42
Omitting Costenbader et al(NHSII)	1.10	0.83	1.46
Vitamin E			
Included all studies	0.84	0.69	1.03
Omitting Cerhan et al(IWHS)	0.84	0.67	1.05
Omitting Costenbader et al(NHS)	0.92	0.66	1.28
Omitting Costenbader et al(NHSII)	0.81	0.65	1.01
α-carotenoid			
Included all studies	1.02	0.78	1.35
Omitting Costenbader et al(NHS)	0.88	0.61	1.26
Omitting Costenbader et al(NHSII)	1.14	0.90	1.45
Omitting Cerhan et al(IWHS)	0.97	0.58	1.62
β-carotenoid			
Included all studies	1.06	0.79	1.43
Omitting Costenbader et al(NHS)	0.88	0.64	1.23
Omitting Costenbader et al(NHSII)	1.18	0.91	1.55
Omitting Cerhan et al(IWHS)	1.06	0.66	1.69
β-cryptoxanthin			
Included all studies	0.86	0.58	1.26
Omitting Costenbader et al(NHS)	0.71	0.47	1.07

Omitting Costenbader et al(NHSII)	0.82	0.45	1.52
Omitting Cerhan et al(IWHS)	1.06	0.84	1.33
Lycopene			
Included all studies	0.97	0.77	1.21
Omitting Costenbader et al(NHS)	0.94	0.61	1.65
Omitting Costenbader et al(NHSII)	0.91	0.71	1.18
Omitting Cerhan et al(IWHS)	1.05	0.81	1.37
Lutein/zeaxanthin			
Included all studies	0.97	0.77	1.23
Omitting Costenbader et al(NHS)	0.83	0.59	1.16
Omitting Costenbader et al(NHSII)	1.04	0.83	1.32
Omitting Cerhan et al(IWHS)	0.94	0.61	1.43

[1]Effect sizes were pooled using random-effects model.

Table S12 (a). Trim and fill tests for food group consumption and risk of rheumatoid arthritis

Food exposure	n to trim	Estimated effect after adding hypothesized studies
Total alcohol	0	0.76(0.68-0.85)
Total alcohol*	2	0.88(0.77,0.99)
Wine	2	0.99(0.84-1.17)
Beer	0	0.87(0.74-1.02)
Liquor	2	0.94(0.80-1.12)
Total coffee	3	1.03(0.84,1.25)
Caffeinated coffee	0	1.30(1.09,1.54)
Decaffeinated coffee	1	1.16(0.77,1.75)
Caffeine	0	1.24(0.93,1.64)
Tea	1	1.21(1.10,1.32)
Fruit	0	0.88(0.79,0.97)
Vegetable	0	0.90(0.76,1.06)
Total meat	1	0.91(0.81,1.03)
Red meat	2	1.13(0.98,1.30)
Poultry	0	1.11(0.95,1.28)
Total fish	0	0.92(0.78,1.08)
Total dairy	0	0.97(0.72,1.30)
Cereal product	0	0.87(0.80,0.96)

*seropositive RA

Table S12 (b). Trim and fill tests for nutrient intake and risk of rheumatoid arthritis

Nutrients exposure	n to trim	Estimated effect after adding hypothesized studies
N-3 PUFA	2	1.01(0.82,1.24)
Vitamin C	0	0.98(0.76,1.27)
Vitamin D	1	0.74(0.59,0.93)
Vitamin D supplement	2	0.66(0.31,1.42)
Vitamin E	2	0.80(0.67,0.95)

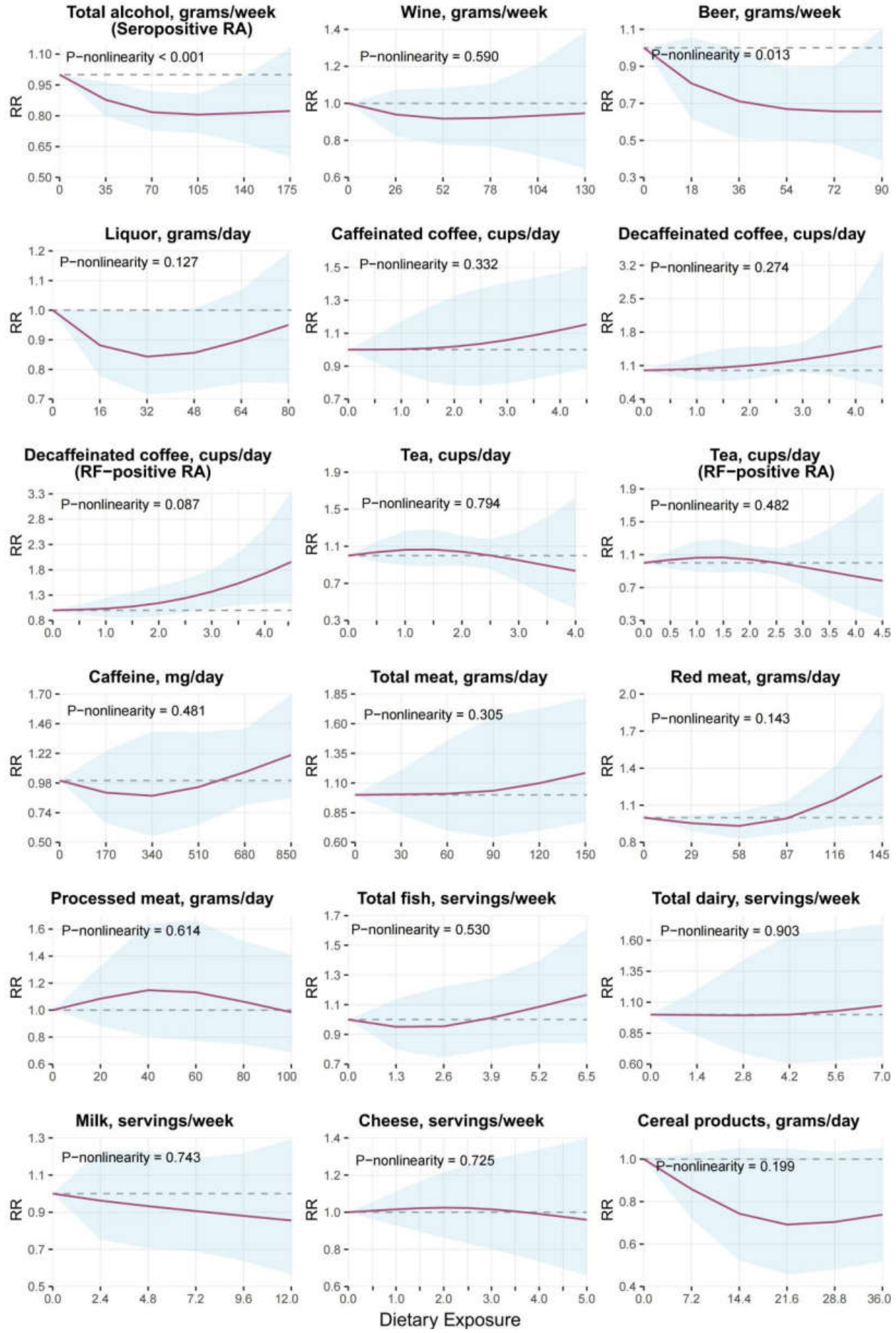

Figure S1. Non-linear dose-response meta-analyses for consumption of food group, and beverages and risk of rheumatoid arthritis using restricted cubic splines.

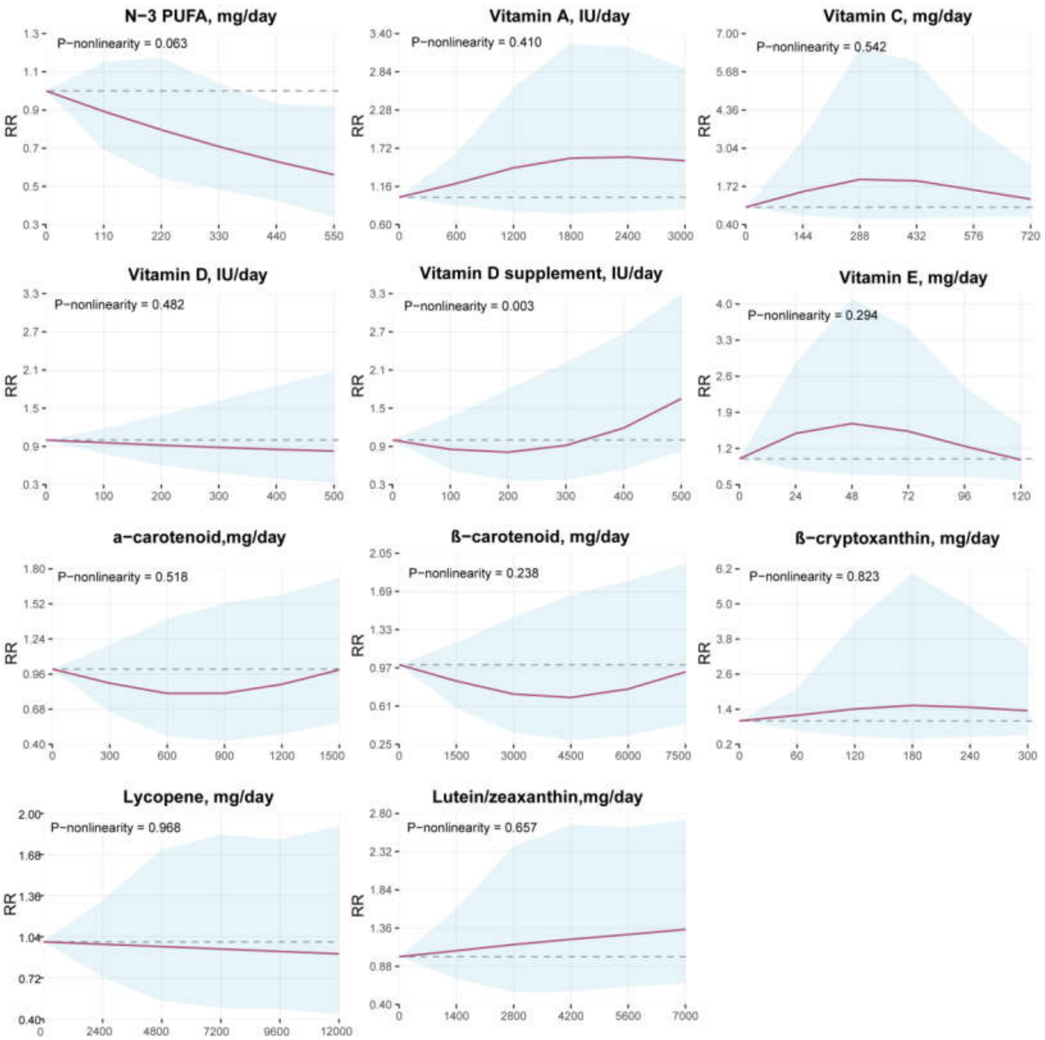

Figure S2. Non-linear dose-response meta-analyses for nutrient intake and risk of rheumatoid arthritis using restricted cubic splines.

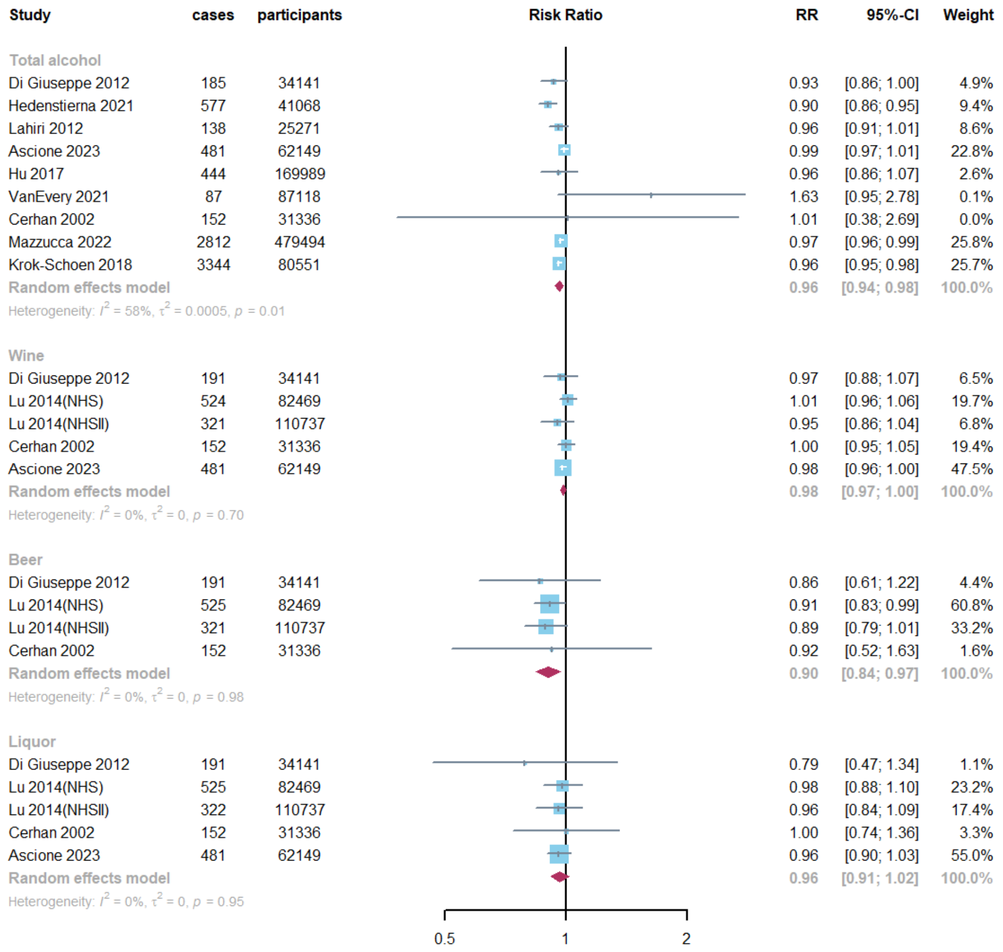

Figure S3. Linear dose-response analyses for alcohol consumption and risk of rheumatoid arthritis

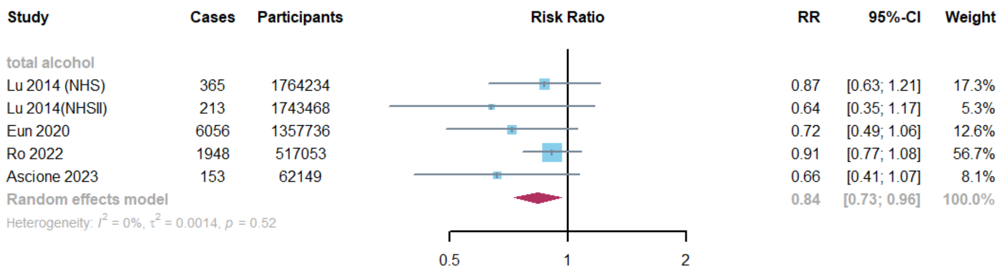

Figure S4. Meta-analysis of total alcohol consumption and risk of seropositive rheumatoid arthritis comparing highest with the lowest categories

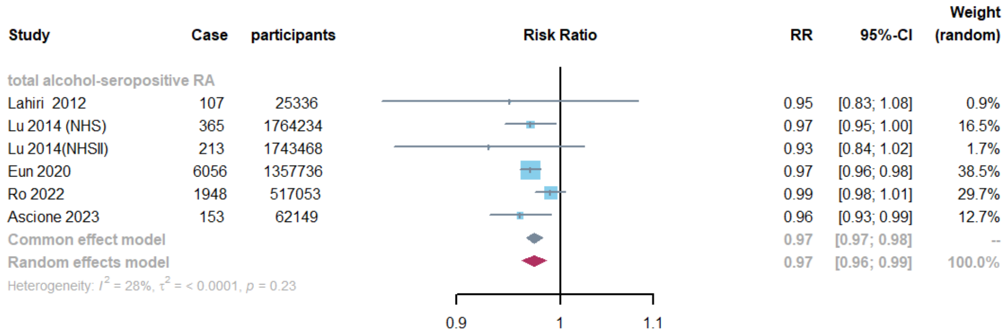

Figure S5. Linear dose-response analyses for total alcohol consumption and risk of seropositive rheumatoid arthritis

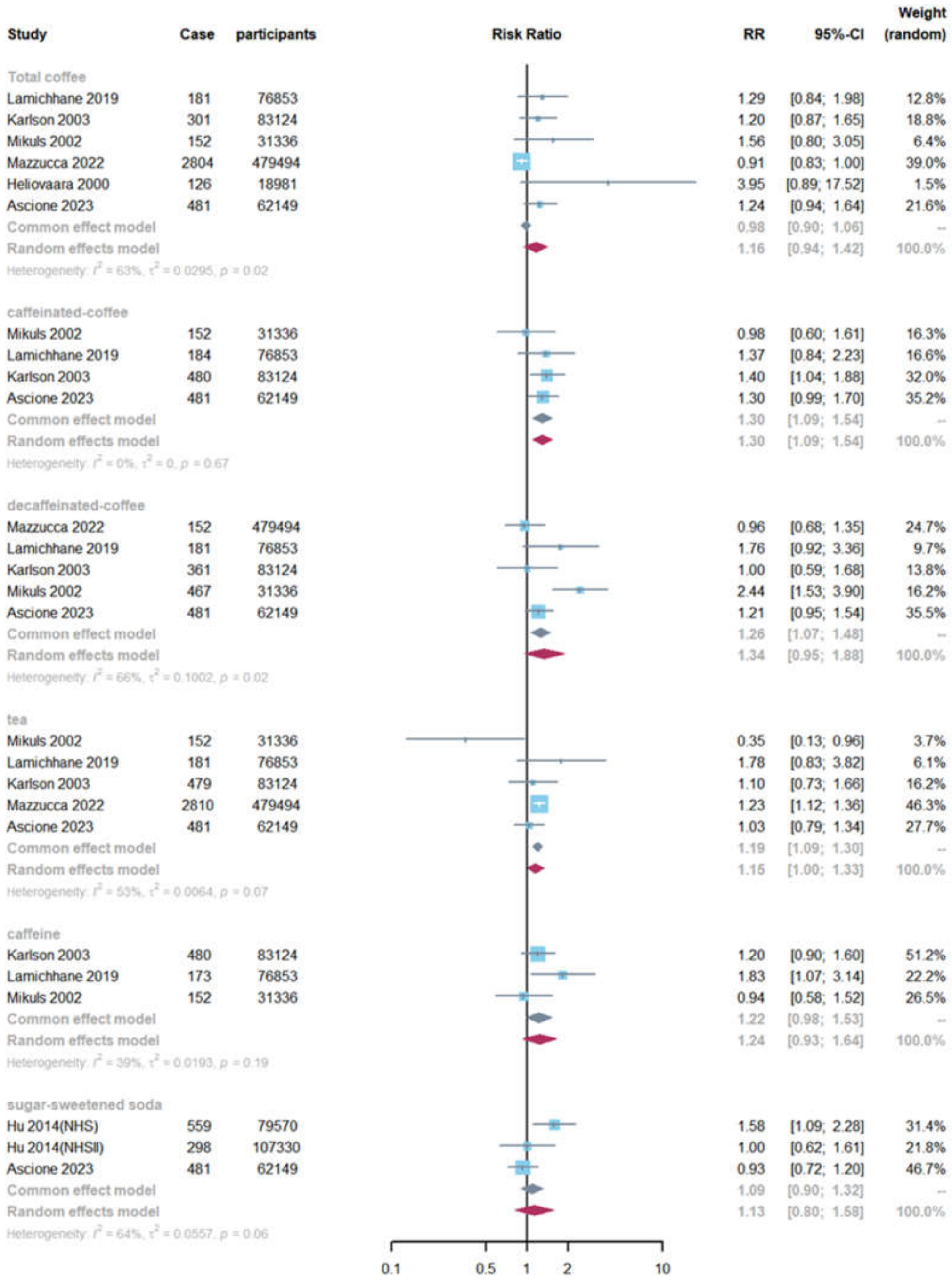

Figure S6. Meta-analysis of non-alcoholic beverages (tea and coffee, soda) consumption and risk of rheumatoid arthritis comparing highest with the lowest categories

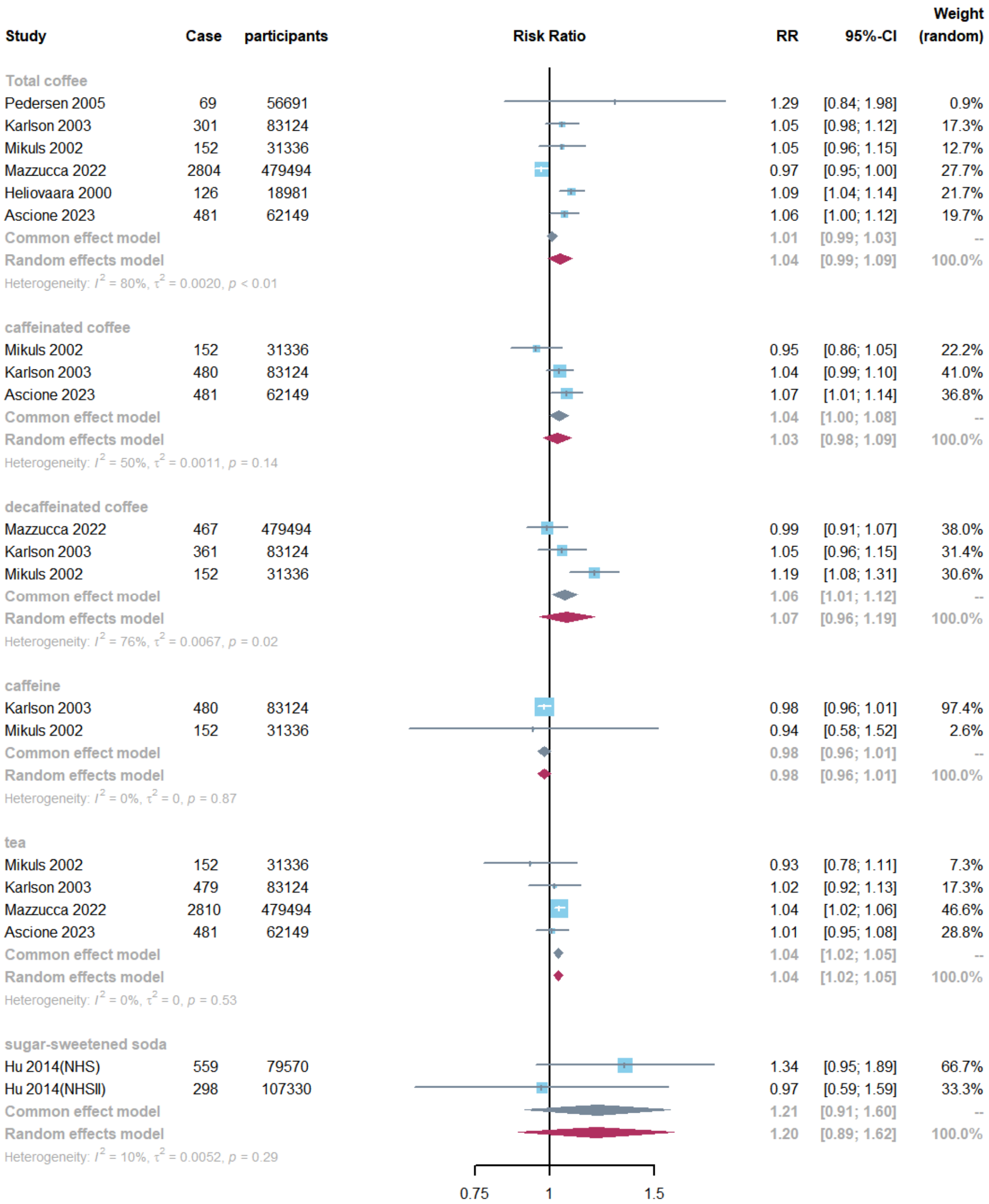

Figure S7. Linear dose-response analyses for non-alcoholic beverages (tea, coffee, and soda) consumption and risk of rheumatoid arthritis

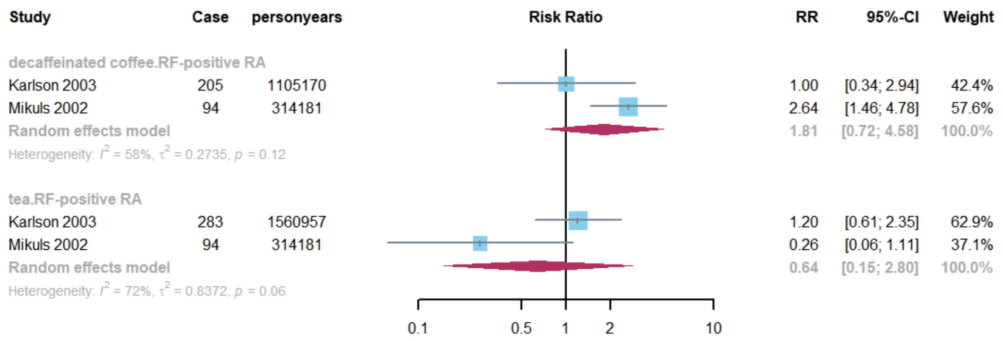

Figure S8. Meta-analysis of decaffeinated coffee, and tea consumption and risk of RF-positive rheumatoid arthritis comparing highest with the lowest categories

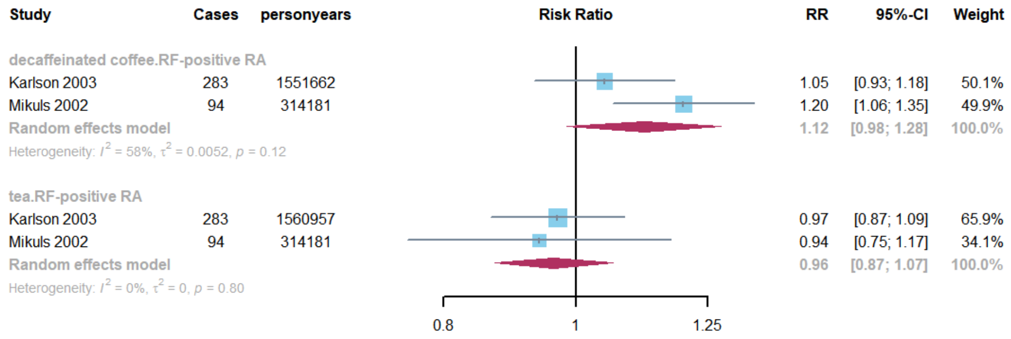

Figure S9. Linear dose-response analyses for decaffeinated coffee, and tea consumption and risk of RF-positive rheumatoid arthritis

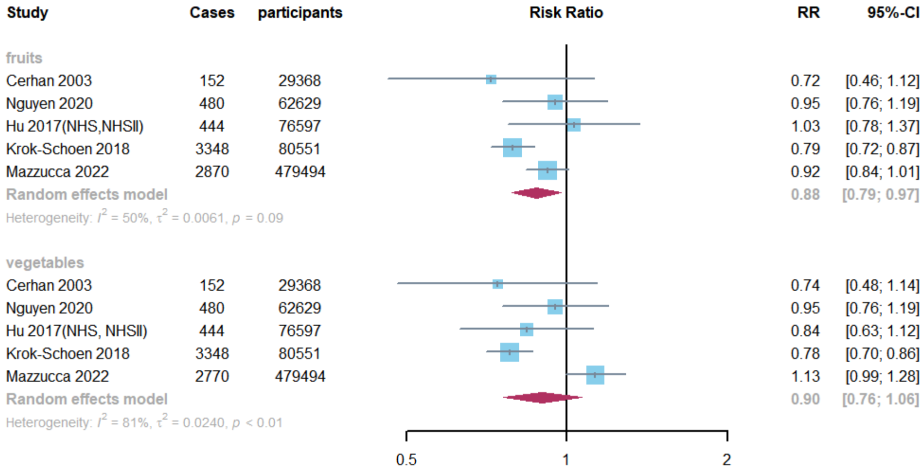

Figure S10. Meta-analysis of fruit, and vegetable consumption and risk of rheumatoid arthritis comparing highest with the lowest categories

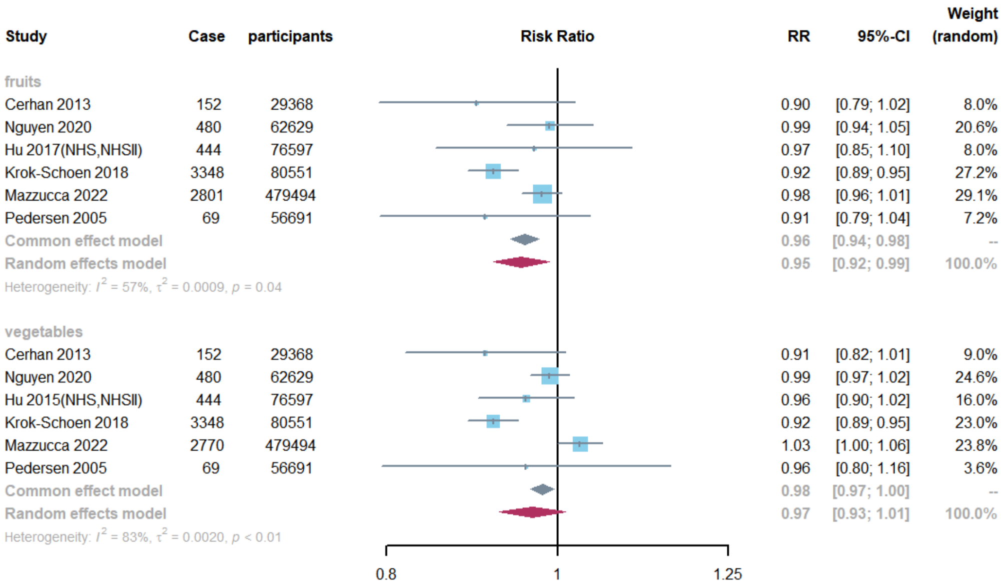

Figure S11. Linear dose-response analyses for fruit, and vegetable consumption and risk of RA

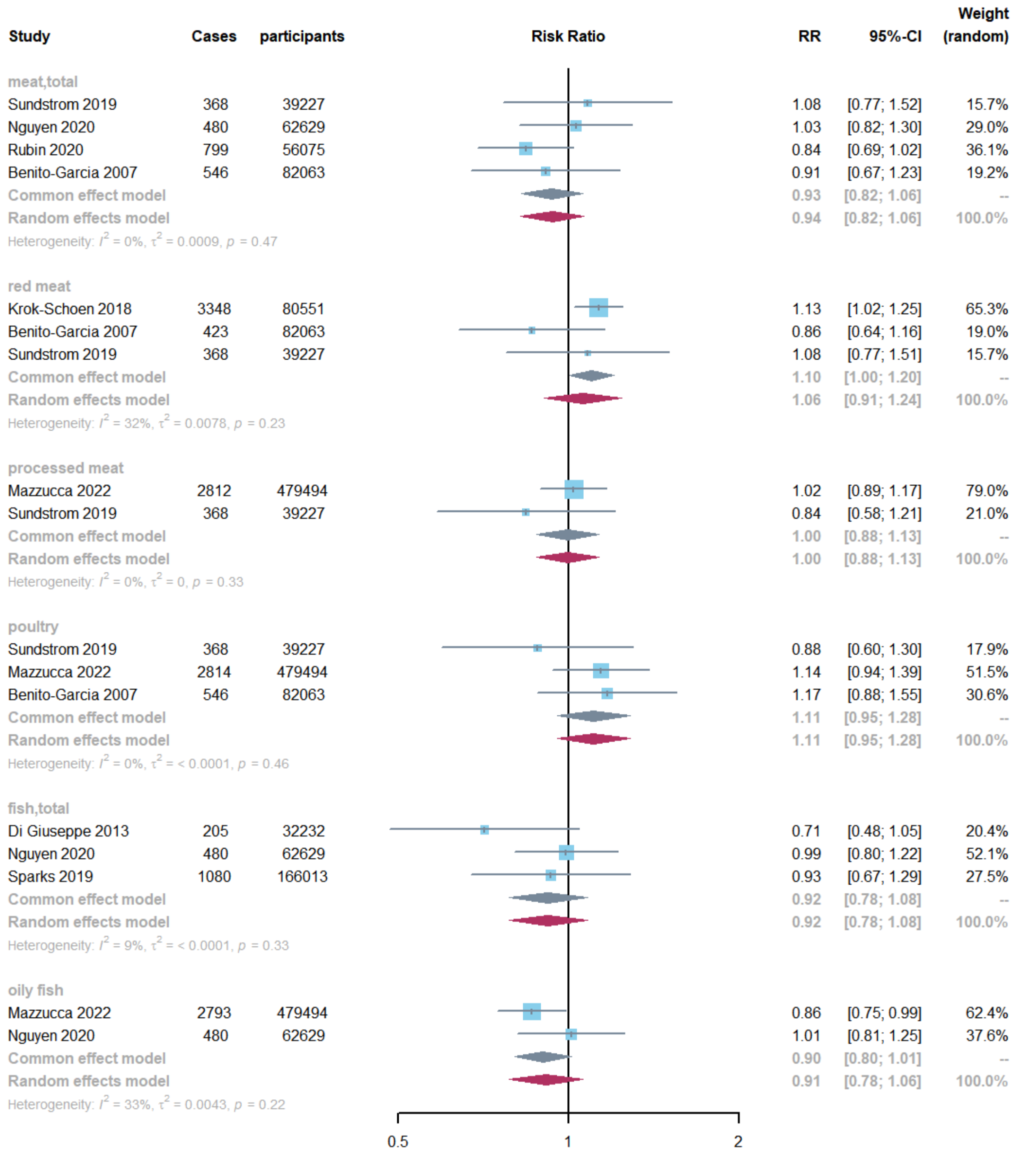

Figure S12. Meta-analysis of meat, and fish consumption and risk of rheumatoid arthritis comparing highest with the lowest categories

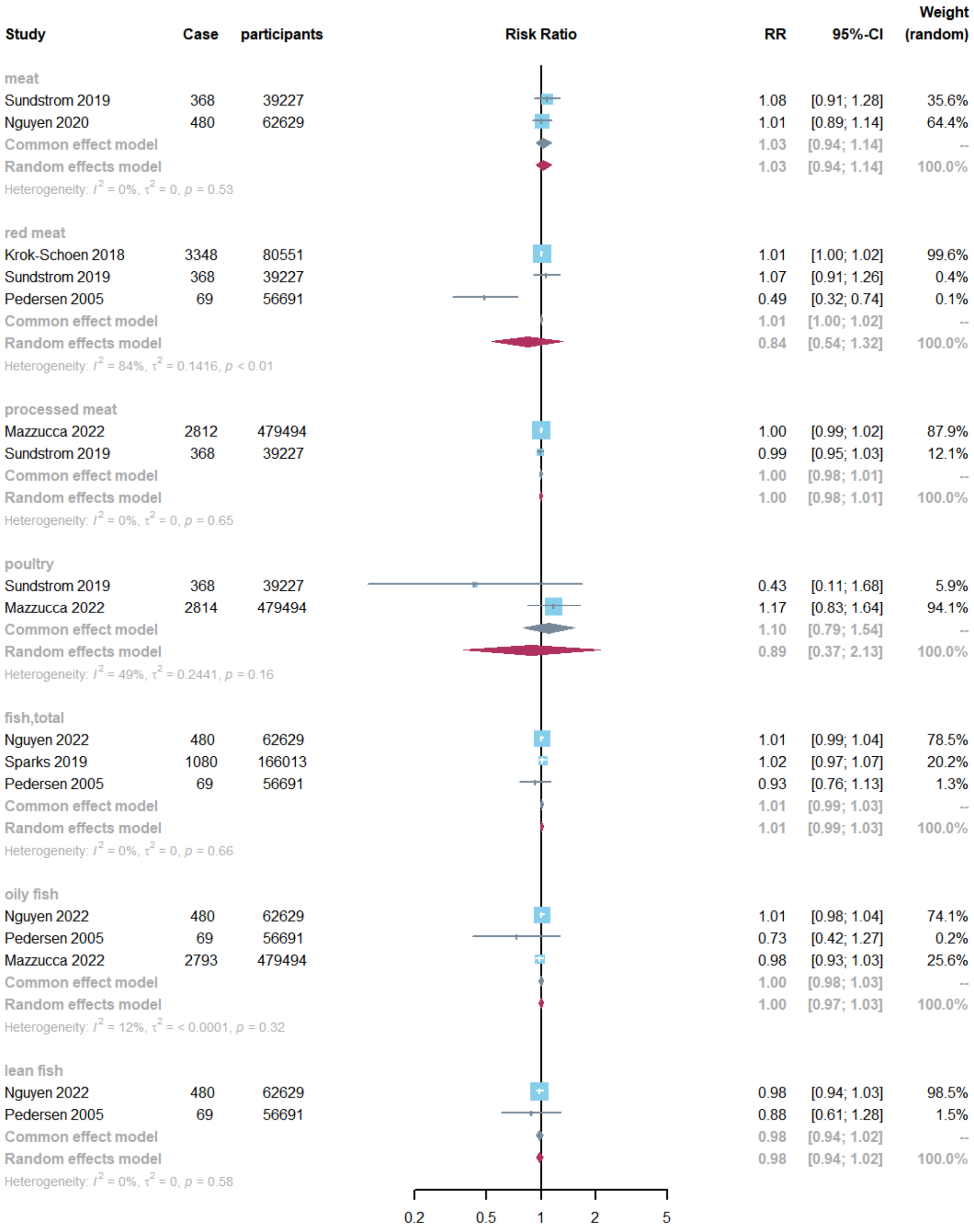

Figure S13. Linear dose-response analyses for meat, and fish consumption and risk of rheumatoid arthritis

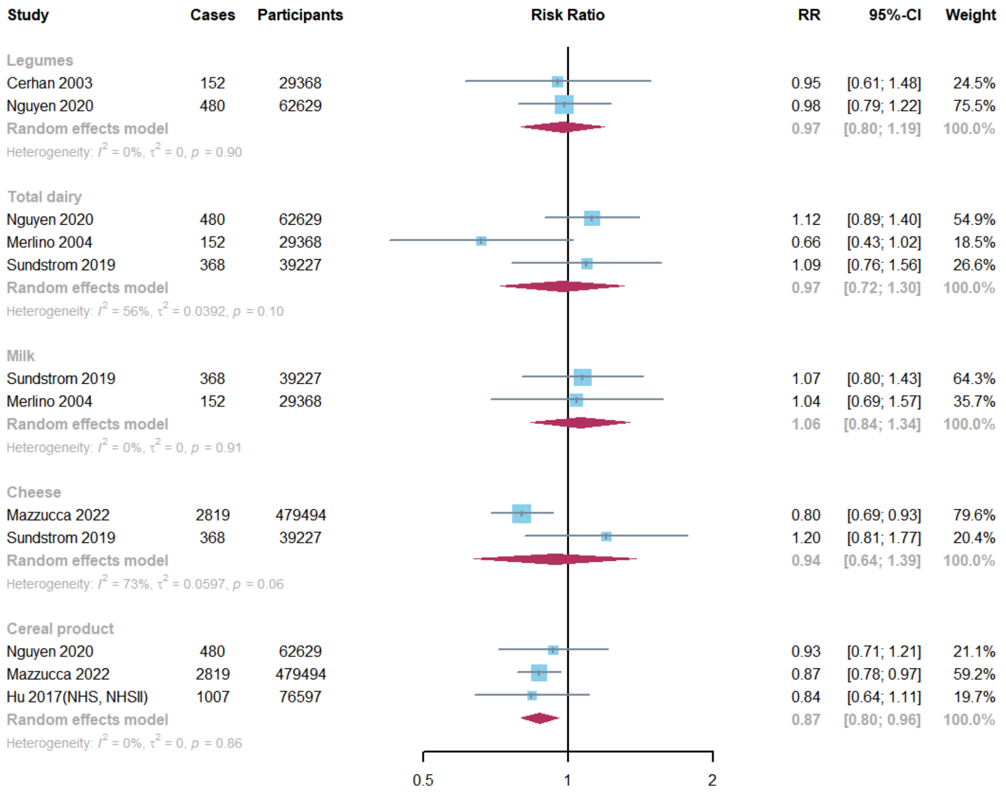

Figure S14. Meta-analysis of other food group consumption and risk of rheumatoid arthritis comparing highest with the lowest categories

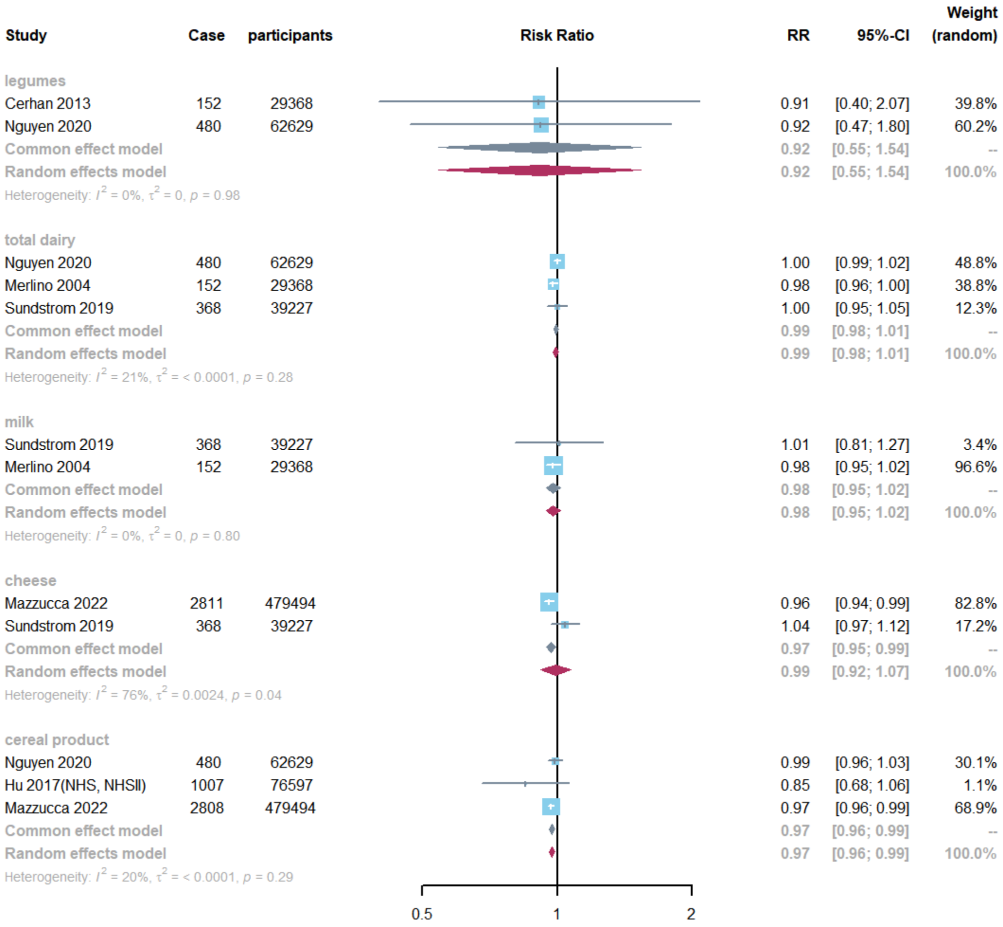

Figure S15. Linear dose-response analyses for other food group consumption and risk of rheumatoid arthritis

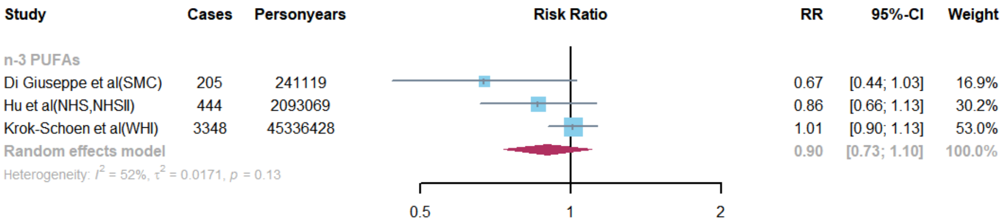

Figure S16. Meta-analysis of dietary n-3 PUFA and risk of rheumatoid arthritis comparing highest with the lowest categories

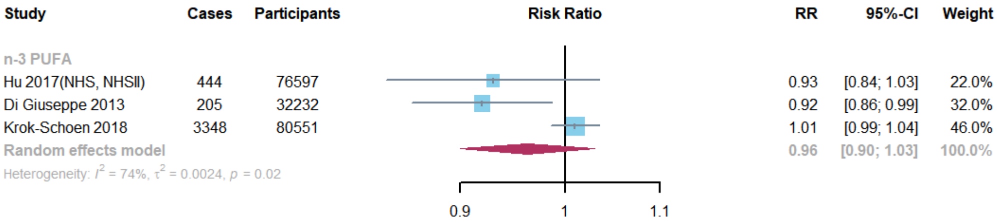

Figure S17. Linear dose-response analyses for dietary n-3 PUFA and risk of rheumatoid arthritis

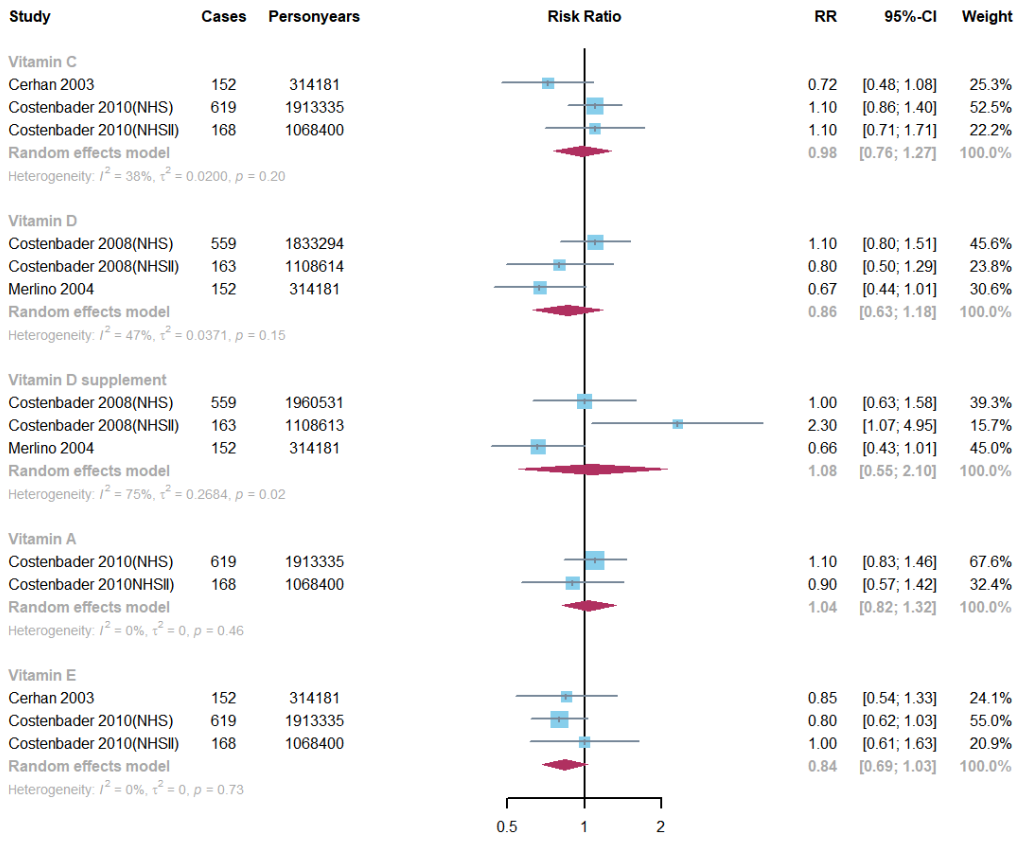

Figure S18. Meta-analysis of vitamin intake and risk of rheumatoid arthritis comparing highest with the lowest categories

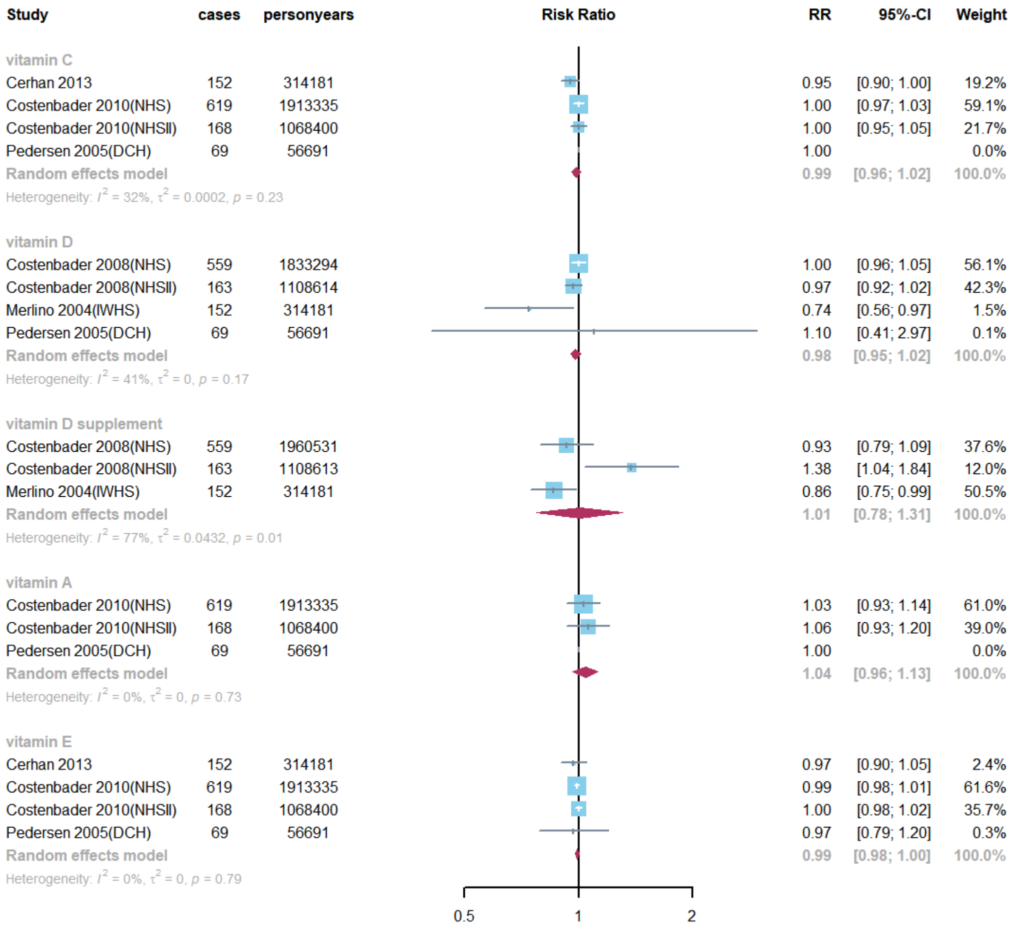

Figure S19. Linear dose-response analyses for vitamins intake and risk of rheumatoid arthritis

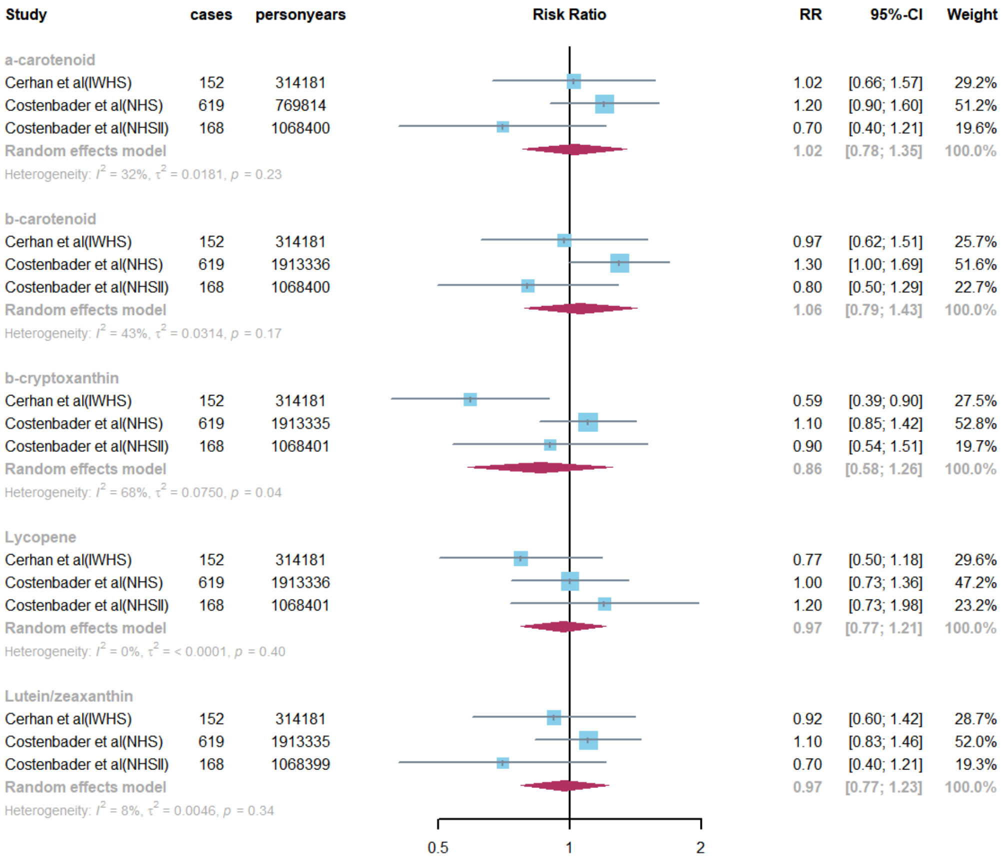

Figure S20. Meta-analysis of dietary carotenoids intake and the risk of rheumatoid arthritis comparing highest with the lowest categories

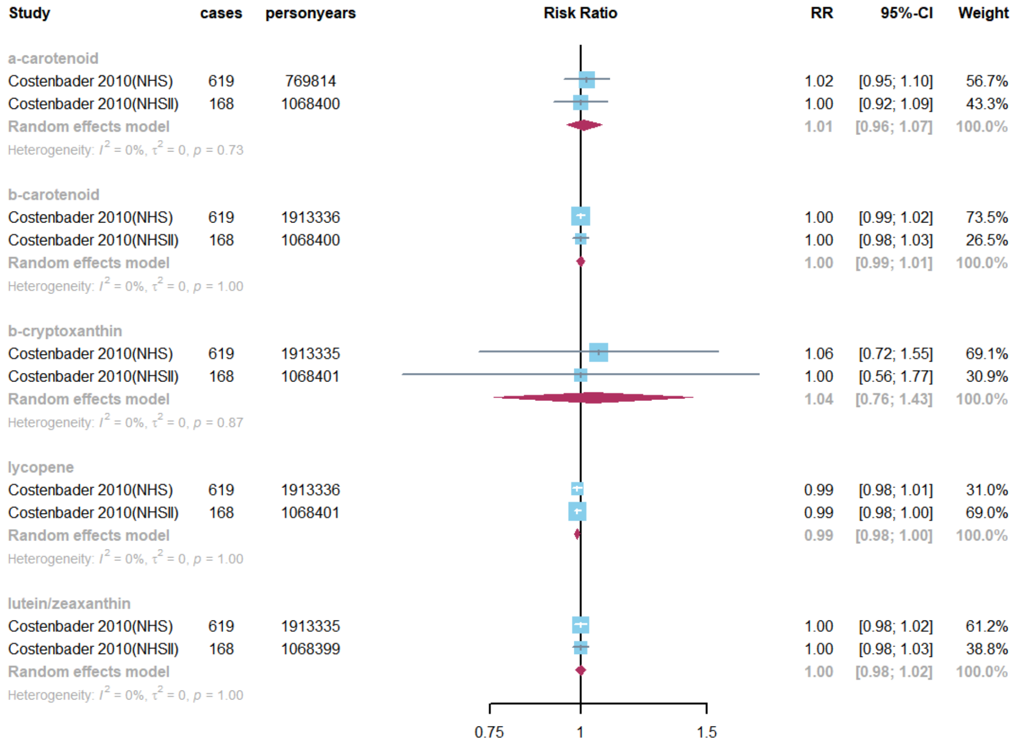

Figure S21. Linear dose-response analyses for dietary carotenoids intake and risk of rheumatoid arthritis

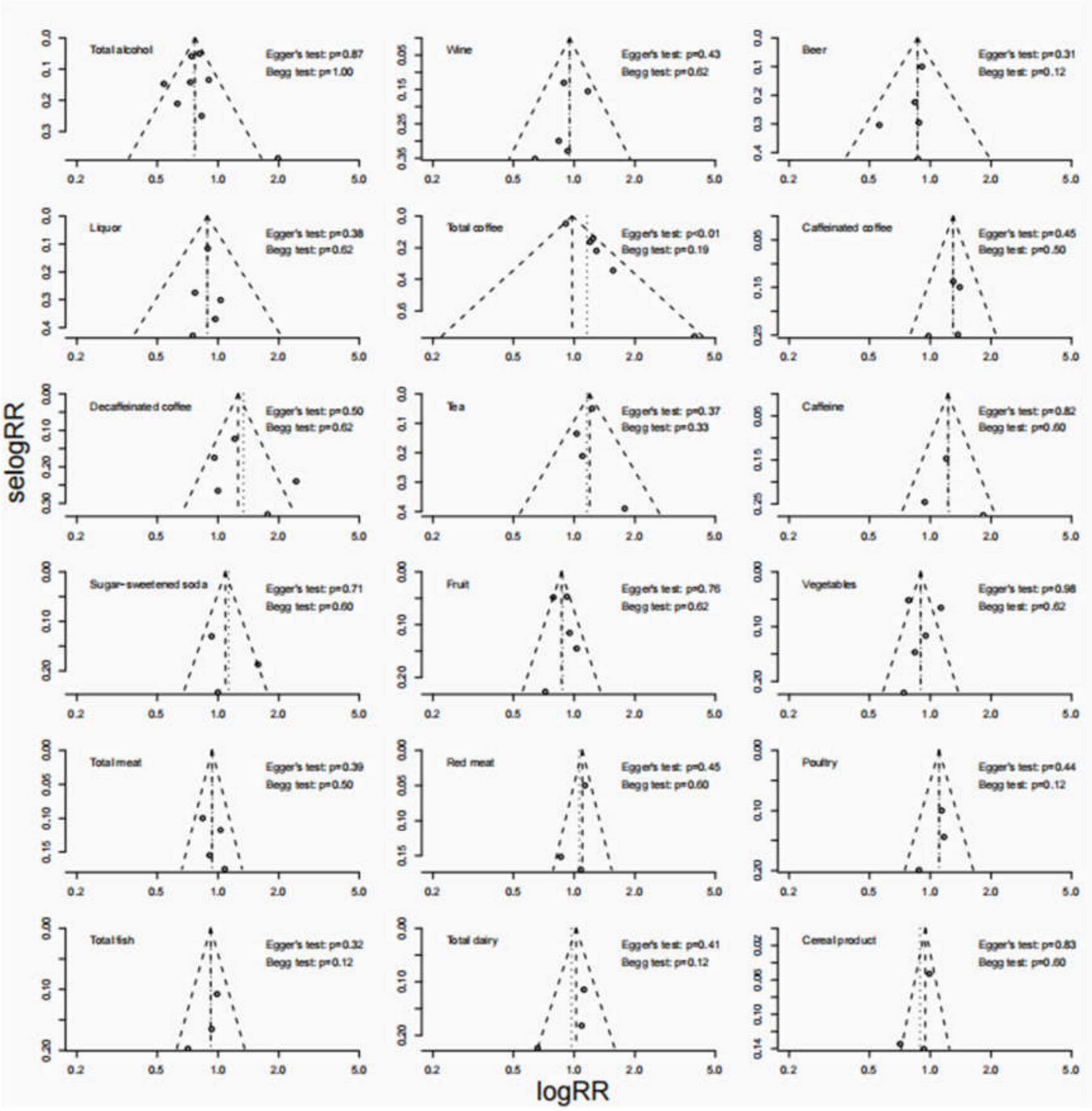

Figure S22. Funnel plots for food group, and beverages consumption and risk of rheumatoid arthritis

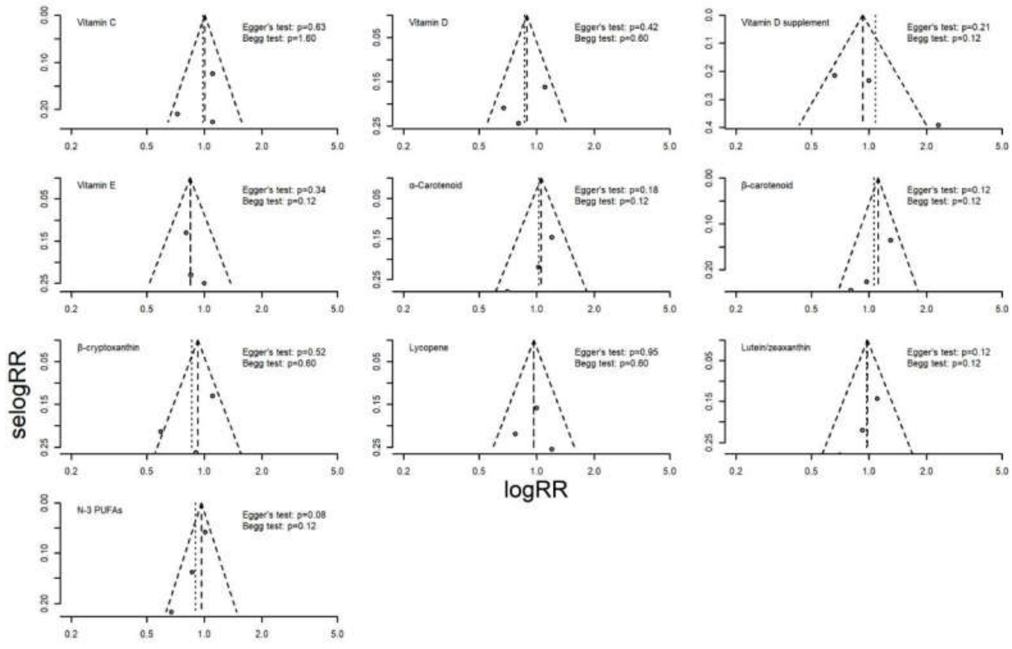

Figure S23. Funnel plots for nutrients intake and risk of rheumatoid arthritis

References

1. Cerhan JR, Saag KG, Merlino LA, et al. Antioxidant micronutrients and risk of rheumatoid arthritis in a cohort of older women. *American journal of epidemiology* 2003;157(4):345-54. doi: 10.1093/aje/kwf205 [published Online First: 2003/02/13]
2. Costenbader KH, Feskanich D, Holmes M, et al. Vitamin D intake and risks of systemic lupus erythematosus and rheumatoid arthritis in women. *Annals of the rheumatic diseases* 2008;67(4):530-5. doi: 10.1136/ard.2007.072736 [published Online First: 2007/08/02]
3. Costenbader KH, Kang JH, Karlson EW. Antioxidant intake and risks of rheumatoid arthritis and systemic lupus erythematosus in women. *American journal of epidemiology* 2010;172(2):205-16. doi: 10.1093/aje/kwq089 [published Online First: 2010/06/11]
4. Di Giuseppe D, Alfredsson L, Bottai M, et al. Long term alcohol intake and risk of rheumatoid arthritis in women: a population based cohort study. *BMJ (Clinical research ed)* 2012;345:e4230. doi: 10.1136/bmj.e4230 [published Online First: 2012/07/12]
5. Sundström B, Ljung L, Di Giuseppe D. Consumption of Meat and Dairy Products Is Not Associated with the Risk for Rheumatoid Arthritis among Women: A Population-Based Cohort Study. *Nutrients* 2019;11(11) doi: 10.3390/nu11112825 [published Online First: 2019/11/23]
6. Di Giuseppe D, Crippa A, Orsini N, et al. Fish consumption and risk of rheumatoid arthritis: a dose-response meta-analysis. 2014;16:1-7.
7. Hedenstierna L, Bellocco R, Ye W, et al. Effects of alcohol consumption and smoking on risk for RA: results from a Swedish prospective cohort study. *RMD open* 2021;7(1) doi: 10.1136/rmdopen-2020-001379 [published Online First: 2021/01/09]
8. Hiraki LT, Munger KL, Costenbader KH, et al. Dietary intake of vitamin D during adolescence and risk of adult-onset systemic lupus erythematosus and rheumatoid arthritis. *Arthritis care & research* 2012;64(12):1829-36. doi: 10.1002/acr.21776 [published Online First: 2012/06/30]
9. Merlino LA, Curtis J, Mikuls TR, et al. Vitamin D intake is inversely associated with rheumatoid arthritis: results from the Iowa Women's Health Study. *Arthritis and rheumatism* 2004;50(1):72-7. doi: 10.1002/art.11434 [published Online First: 2004/01/20]
10. Lahiri M, Luben RN, Morgan C, et al. Using lifestyle factors to identify individuals at higher risk of inflammatory polyarthritis (results from the European Prospective Investigation of Cancer-Norfolk and the Norfolk Arthritis Register--the EPIC-2-NOAR Study). *Annals of the rheumatic diseases* 2014;73(1):219-26. doi: 10.1136/annrheumdis-2012-202481 [published Online First: 2013/03/19]
11. Nguyen Y, Salliot C, Gelot A, et al. Mediterranean Diet and Risk of Rheumatoid Arthritis: Findings From the French E3N-EPIC Cohort Study. *Arthritis & rheumatology (Hoboken, NJ)* 2021;73(1):69-77. doi: 10.1002/art.41487 [published Online First: 2020/09/11]
12. Hu Y, Sparks JA, Malspeis S, et al. Long-term dietary quality and risk of developing rheumatoid arthritis in women. *Annals of the rheumatic diseases* 2017;76(8):1357-64. doi: 10.1136/annrheumdis-2016-210431 [published Online First: 2017/02/01]
13. Karlson EW, Mandl LA, Aweh GN, et al. Coffee consumption and risk of rheumatoid arthritis. *Arthritis and rheumatism* 2003;48(11):3055-60. doi: 10.1002/art.11306 [published Online First: 2003/11/13]
14. Krok-Schoen JL, Brasky TM, Hunt RP, et al. Dietary Long-Chain n-3 Fatty Acid Intake and Arthritis Risk in the Women's Health Initiative. *Journal of the Academy of Nutrition and Dietetics* 2018;118(11):2057-69. doi: 10.1016/j.jand.2018.04.005 [published Online First: 2018/06/21]
15. Lamichhane D, Collins C, Constantinescu F, et al. Coffee and Tea Consumption in Relation to Risk of Rheumatoid Arthritis in the Women's Health Initiative Observational Cohort. *Journal of clinical rheumatology : practical reports on rheumatic & musculoskeletal diseases* 2019;25(3):127-32. doi: 10.1097/rhu.0000000000000788 [published Online First: 2018/05/26]
16. Lu B, Solomon DH, Costenbader KH, et al. Alcohol consumption and risk of incident rheumatoid arthritis in women: a prospective study. *Arthritis & rheumatology (Hoboken, NJ)* 2014;66(8):1998-2005. doi: 10.1002/art.38634 [published Online First: 2014/04/15]
17. Mikuls TR, Cerhan JR, Criswell LA, et al. Coffee, tea, and caffeine consumption and risk of rheumatoid arthritis: results from the Iowa Women's Health Study. *Arthritis and rheumatism* 2002;46(1):83-91. doi: 10.1002/1529-0131(200201)46:1<83::Aid-art10042>3.0.Co;2-d [published Online First: 2002/01/31]
18. Pedersen M, Stripp C, Klarlund M, et al. Diet and risk of rheumatoid arthritis in a prospective cohort. *The Journal of rheumatology* 2005;32(7):1249-52. [published Online First: 2005/07/05]
19. Rubin KH, Rasmussen NF, Petersen I, et al. Intake of dietary fibre, red and processed meat and risk of late-onset Chronic Inflammatory Diseases: A prospective Danish study on the "diet, cancer

and health" cohort. *International journal of medical sciences* 2020;17(16):2487-95. doi: 10.7150/ijms.49314 [published Online First: 2020/10/09]

20. Sparks JA, O'Reilly É J, Barbhaiya M, et al. Association of fish intake and smoking with risk of rheumatoid arthritis and age of onset: a prospective cohort study. *BMC musculoskeletal disorders* 2019;20(1):2. doi: 10.1186/s12891-018-2381-3 [published Online First: 2019/01/07]
21. VanEvery H, Yang W, Olsen N, et al. Alcohol Consumption and Risk of Rheumatoid Arthritis among Chinese Adults: A Prospective Study. *Nutrients* 2021;13(7) doi: 10.3390/nu13072231 [published Online First: 2021/07/03]
22. Cerhan JR, Saag KG, Criswell LA, et al. Blood transfusion, alcohol use, and anthropometric risk factors for rheumatoid arthritis in older women. *The Journal of rheumatology* 2002;29(2):246-54. [published Online First: 2002/02/13]
23. Benito-Garcia E, Feskanich D, Hu FB, et al. Protein, iron, and meat consumption and risk for rheumatoid arthritis: a prospective cohort study. *Arthritis Res Ther* 2007;9(1):R16. doi: 10.1186/ar2123 [published Online First: 2007/02/10]
24. Hu Y, Costenbader KH, Gao X, et al. Sugar-sweetened soda consumption and risk of developing rheumatoid arthritis in women. *The American journal of clinical nutrition* 2014;100(3):959-67. doi: 10.3945/ajcn.114.086918 [published Online First: 2014/07/18]
25. Nguyen Y, Salliot C, Mariette X, et al. Fish Consumption and Risk of Rheumatoid Arthritis: Findings from the E3N Cohort Study. *Nutrients* 2022;14(4) doi: 10.3390/nu14040861 [published Online First: 2022/02/27]
26. Heliövaara M, Aho K, Knekt P, et al. Coffee consumption, rheumatoid factor, and the risk of rheumatoid arthritis. *Annals of the rheumatic diseases* 2000;59(8):631-5. doi: 10.1136/ard.59.8.631 [published Online First: 2000/07/27]
27. Mazzucca CB, Scotti L, Cappellano G, et al. Nutrition and Rheumatoid Arthritis Onset: A Prospective Analysis Using the UK Biobank. *Nutrients* 2022;14(8) doi: 10.3390/nu14081554 [published Online First: 2022/04/24]
28. Eun Y, Jeon KH, Han K, et al. Menopausal factors and risk of seropositive rheumatoid arthritis in postmenopausal women: a nationwide cohort study of 1.36 million women. *Scientific reports* 2020;10(1):20793. doi: 10.1038/s41598-020-77841-1 [published Online First: 2020/11/29]
29. Ro J, Kim SH, Kim HR, et al. Impact of lifestyle and comorbidities on seropositive rheumatoid arthritis risk from Korean health insurance data. *Scientific reports* 2022;12(1):2201. doi: 10.1038/s41598-022-06194-8 [published Online First: 2022/02/11]
30. Ascione S, Barde F, Artaud F, et al. Association between beverage consumption and risk of rheumatoid arthritis: a prospective study from the French E3N Cohort. *Rheumatology (Oxford, England)* 2023;62(5):1814-23. doi: 10.1093/rheumatology/keac544 [published Online First: 2022/10/01]
